# LenRuler: a rice-centric method for automated radicle length measurement with multicrop validation

**DOI:** 10.1016/j.plaphe.2025.100103

**Published:** 2025-09-08

**Authors:** Jinfeng Zhao, Zeyu Hou, Hua Hua, Qianlong Nie, Yuqian Pang, Yan Ma, Xuehui Huang

**Affiliations:** a Shanghai Key Laboratory of Plant Molecular Sciences, College of Life Sciences, Shanghai Normal University, Shanghai, 200234, China; b College of Information, Mechanical and Electrical Engineering, Shanghai Normal University, Shanghai, 200234, China

**Keywords:** Rice, Radicle length, Automated measurement, Image segmentation, Multicrop validation

## Abstract

Radicle length is a critical indicator of seed vigor, germination capacity, and seedling growth potential. However, existing measurement methods face challenges in automation, efficiency, and generalizability, often requiring manual intervention or re-annotation for different seed types. To address these limitations, this paper proposes an automated method, LenRuler, with a primary focus on rice seeds and validation in multiple crops. The method leverages the Segment Anything Model (SAM) as the foundational segmentation model and employs a coarse-to-fine segmentation strategy combined with Gaussian-based classification to automatically generate bounding boxes and centroids, which are then fed into SAM for precise segmentation of the seed coat and radicle. The radicle length is subsequently computed by converting the geodesic distance between the radicle skeleton's farthest endpoint and its nearest intersection with the seed coat skeleton into the true length. Experiments on the Riceseed1 dataset show that the proposed method achieves a Dice coefficient of 0.955 and a Pixel Accuracy of 0.944, demonstrating excellent segmentation performance. Radicle length measurement experiments on the Riceseed2 test set show that the Mean Absolute Error (MAE) was 0.273 ​mm and the coefficient of determination (R^2^) was 0.982, confirming the method's high precision for rice. On the Otherseed dataset, the predicted radicle lengths for maize (*Zea mays*), pearl millet (*Pennisetum glaucum*), and rye (*Secale cereale*) are consistent with the observed radicle length distributions, demonstrating strong cross-species performance. These results establish LenRuler as an accurate and automated solution for radicle length measurement in rice, with validated applicability to other crop species.

## 1 Introduction

Seeds are the foundation of agricultural production, and their quality directly affects the yield, quality, and growth potential of crops. As the foundation for crop growth and development, seed quality plays an irreplaceable role in agricultural production efficiency and sustainable development. In the field of seed science, seed phenotype is an important indicator of seed quality, covering various aspects such as seed vigor, purity, and health status [1,2]. Precise measurement of seed phenotypes enables breeders to select high-quality genetic resources. This, in turn, facilitates advancements in precision agriculture [[3], [4], [5]].

Among various seed phenotypic parameters, radicle or plumule length has gained increasing attention due to its strong correlation with seed vigor, germination capacity, and seedling growth potential. The length of the radicle and plumule is crucial for selecting high-vigor seeds, and it also provides important insights for gene function studies, improving agricultural productivity, and formulating breeding strategies. For instance, in investigations of seed vigor in rice, the length of the radicle or plumule has been employed to validate the regulatory role of the *OsJMJ718* gene in seed germination and seedling development [6]. Additionally, researchers have leveraged this phenotypic parameter to assess the impact of the *GF14h* gene on the early growth of rice seeds, facilitating the selection of varieties that can germinate under diverse temperature conditions (such as high or low temperatures), thereby addressing the challenges posed by global climate change [7]. Therefore, radicle or plumule length serves not only as a vital tool for uncovering the intrinsic vigor of seeds but also as a crucial scientific foundation for seed selection and agricultural production [8,9].

In seed germination studies, seeds are typically immersed in water to facilitate treatment. Under these conditions, the radicle is the first structure to emerge. When the radicle penetrates the seed coat and reaches a length of 1–2 ​mm, the seed is generally classified as having germinated [10,11]. Accurate measurement of radicle length at this critical stage is essential for evaluating seed vigor and subsequent growth potential. Consequently, this study focuses on the automated measurement of radicle length. To achieve this objective, precise measuring instruments, such as millimeter rulers or calipers, can be employed. Given that the radicle may exhibit curvature during growth, it is necessary to straighten it prior to measurement [12,13]. Currently, software applications such as ImageJ [14] and GERMINATOR [15] are extensively utilized for radicle length assessment. In the case of ImageJ, the methodology involves manually marking the distance from the seed coat to the tip of the radicle and converting this marked length into the actual measurement. This approach, which relies on manual annotations, is unaffected by variables such as seed color, thereby making it applicable to various seed types, including Striga hermonthica [16], Arabidopsis thaliana [17], and tomato [18].

In recent years, deep learning and object detection technologies have found widespread applications in the field of agriculture. Several studies have begun to explore the detection and measurement of radicles using deep learning models. For instance, researchers have developed YOLO-r [10] and SGR-YOLO [19] by improving the YOLO (You Only Look Once) framework, enabling the detection of germination status in rice seeds. SeedQuant [20], based on the Faster R-CNN model, has successfully implemented radicle detection. Additionally, DDST-CenterNet [21] improved the CenterNet model for detecting whether seeds have germinated. While these methods excel in rapidly determining seed germination status, they fall short in accurately measuring radicle length. Another approach employs convolutional neural networks to extract features related to internal biological and physiological changes in seed images, thus classifying high-quality seeds from low-quality ones [22]. However, this method also fails to automatically obtain radicle length, necessitating manual measurement. To achieve automated measurement of radicle length, YOLOv8-segANDcal [23] utilizes the segmentation module of YOLOv8 [24] to segment the radicles of soybean seeds and estimates the actual length by calculating half of the radicle contour length. A notable limitation of the YOLOv8-segANDcal method is its dependence on extensive manual annotation of seed masks.

Significant progress has been made in automated root phenotyping by reducing manual intervention and enhancing cross-species adaptability. Regression-based convolutional neural network (CNN) frameworks (e.g., Khoroshevsky et al. [25]) enable image-level trait estimation without requiring segmentation; however, they often lack the precision necessary for measuring fine-scale root substructures. Segmentation-free tools such as AutoRoot [26], which employ Dijkstra's algorithm for probabilistic root tracing, demonstrate potential but are constrained by the need for controlled imaging conditions. High-throughput platforms like BRAT [27] integrate automated plate imaging with skeletonization for extracting Arabidopsis primary roots, yet their reliance on hypocotyl identification limits applicability during early germination stages. MyROOT [28] addresses this by introducing semi-automated hypocotyl-root junction detection with subpixel accuracy, although it does not support branch-specific analysis. Traditional pipelines (e.g., NIH Image [29]) remain in use for root diameter estimation via iterative intercept-length computation, but suffer from orientation-dependent inaccuracies, with reported accuracy ranging between 89 ​% and 126 ​%. Recent advances such as SAM-based methods [30] have shown promise in multi-species segmentation, and MyROOT 2.0 [31] further improves robustness through ridge detection and adaptive edge deletion. Nevertheless, a critical gap remains: the lack of a segmentation-light, annotation-efficient framework for species-agnostic fine-root phenotyping—particularly for capturing curved root structures in complex and heterogeneous germination environments.

Based on the analysis presented, existing methodologies for measuring radicle length encounter several critical limitations. Firstly, the use of software packages such as ImageJ necessitates substantial manual intervention, which not only reduces efficiency but also introduces the potential for measurement errors attributable to human handling. Secondly, while contemporary deep learning models demonstrate strong performance in tasks like seed germination detection and classification, they remain inadequate for the automated measurement of radicle length. Moreover, even when a specialized segmentation model is developed for a specific seed type, its applicability across different varieties is often limited. This necessitates the re-annotation of target masks when applied to other seed types, a process that is both labor-intensive and time-consuming. Consequently, current methods reveal significant shortcomings in terms of automation, efficiency, and general applicability.

This paper presents LenRuler, an automated method for radicle length measurement with robust performance in rice and validated applicability to multiple crop species. The proposed approach iteratively refines the input prompts provided to the segmentation model SAM (Segment Anything Model) [32] by incorporating precise information, including bounding boxes detected by YOLO [33] and the centroid coordinates of the seeds. This process ensures accurate segmentation of the seed coat along with the germination region. Furthermore, leveraging the Gaussian distribution characteristics of the seed coat color, we construct a Gaussian model to effectively classify the segmented components into the seed coat and radicle. Finally, the radicle length is determined by identifying the farthest endpoint from the seed coat skeleton and the nearest intersection point on the radicle. We compute the actual length measurement from the geodesic distance between these two points.

The main contributions of the proposed algorithm are as follows:

1. We introduce an automated seed segmentation approach using SAM, which significantly reduces the reliance on large-scale manual annotation.
2. We utilize YOLO detection bounding boxes and seed centroids as prompt information, inputting them to SAM in a stepwise manner. This approach requires the construction of only the YOLO detection model, as the annotation of bounding boxes is significantly faster than that of segmentation masks.
3. We implement minimal seed imaging requirements, allowing for seed contact during imaging and removing strict constraints on lighting or background, thus enhancing adaptability and robustness.
4. We utilize an alternating segmentation-classification optimization strategy, which iteratively refines segmentation by incorporating informative prompts into SAM, thereby improving segmentation accuracy.

The structure of this paper is arranged as follows: Section 2 introduces the materials and methods used in the study; Section 3 presents the experimental results in detail; Section 4 provides comprehensive discussion, including key contributions and limitations; finally, Section 5 summarizes the paper and provides conclusions.

## 2 Materials and methodology

### 2.1 Materials

This study utilizes three datasets: Riceseed1, Riceseed2, and Otherseed. The details are as follows:

(1) Riceseed1 dataset

Riceseed1 is a publicly available rice seed dataset [10] containing 1,211 images of rice seeds from multiple seed types. The dataset includes a total of 44,660 seeds, among which 28,637 are germinated, and 16,023 are non-germinated. Although this dataset provides a large collection of seed images, it does not contain ground truth measurements of radicle length. Therefore, in this study, Riceseed1 is primarily used for seed segmentation experiments.

For seed germination detection, we employed the pre-trained YOLO-r model [10] directly on Riceseed1 without retraining, leveraging its established capability to distinguish germinated and non-germinated seeds. To develop the segmentation models (UNet [34] and Mask-RCNN [35]), the entire Riceseed1 dataset (1,211 images) was split into training and test sets in an 8:2 ratio. All images were annotated using the LabelMe tool to provide pixel-level masks encompassing both seed bodies and radicles. These annotations served as ground truth for model training and evaluation.

(2) Riceseed2 dataset

To conduct radicle length measurement experiments, we collected 600 images of rice seeds (including both germinated and non-germinated) under the same germination conditions and imaging setup as Riceseed1. Each image contains 15-30 rice seeds, forming the Riceseed2 dataset.

Riceseed2 was split into training and test sets in an 8:2 ratio, and the LabelImg tool was used to annotate germinated and non-germinated seeds. Subsequently, a YOLOv8 model was trained on Riceseed2 for seed detection.

To obtain the true radicle length, two technicians measured all germinated seeds in 120 test images using a vernier caliper with a precision of 0.1 ​mm. These measurements were then verified by a third technician, serving as the ground truth for model evaluation.

(3) Otherseed dataset

To validate the method's adaptability to other crop species, we constructed the Otherseed dataset using germinated seed images from publicly available sources. The dataset consists of the following:

• Maize seed images [36]: A total of 115 images, each containing 35 seeds, resulting in 4,025 seeds. We adopted the same annotation method as Riceseed2 and trained a YOLOv8-based maize seed detection model.
• Pearl millet seed images [37]: A total of 7,954 images, each containing 10 seeds, summing up to 79,540 seeds. This dataset includes pre-existing annotations, allowing direct use in experiments.
• Rye seed images [37]: A total of 7,695 images, each containing 120 seeds. Like the pearl millet dataset, this dataset also provides pre-labeled annotations for model training and evaluation.

Table S1 summarizes the key characteristics of these three datasets, where“-” indicates that this item is not applicable in the experiment.

### 2.2 Methods

#### 2.2.1 Overview

**Fig. 1:**
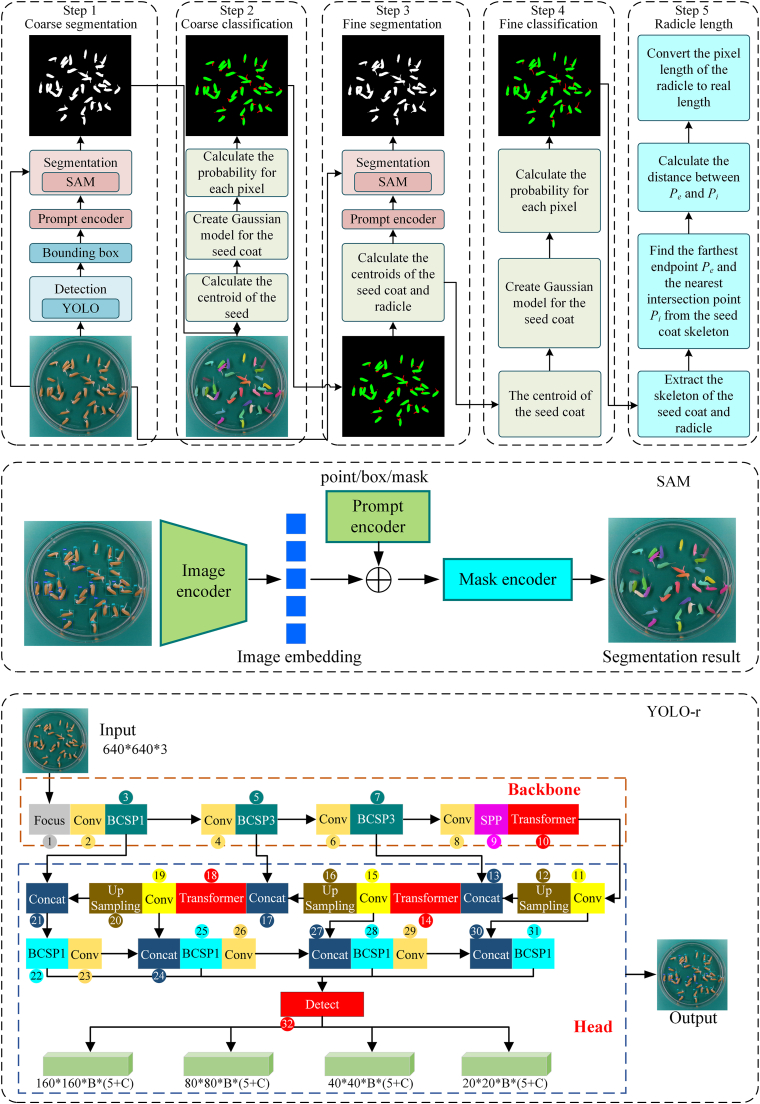
Algorithm overview. The top part illustrates the five steps of LenRuler, the middle section depicts the structure of SAM, and the bottom part shows the architecture of YOLO-r.

Fig. 1 visually integrates the algorithm workflow with detailed architectures of SAM and YOLO-r. As shown in Fig. 1, the proposed algorithm consists of five main steps:

1. Coarse segmentation of seeds: First, YOLO is used to detect germinated and non-germinated seeds in the input image. The bounding boxes generated by YOLO are used as prompts, which, together with the input image, are fed into SAM to perform coarse segmentation for each seed.
2. Coarse classification of seeds: Next, the centroid of each seed is calculated based on the coarse segmentation results. A Gaussian model is established for the seed coat region, which is then used to classify each seed into two categories: seed coat and radicle, completing the coarse classification.
3. Fine segmentation of seeds: Subsequently, the centroids of the seed coat and radicle for each seed are calculated. These centroids are used as prompts, along with the input image, and are fed into SAM to achieve fine segmentation for each seed.
4. Fine classification of seeds: A Gaussian model is re-established based on the seed coat region to further classify the fine segmentation results into two categories: seed coat and radicle.
5. Radicle length calculation: Finally, the skeletons of the seed coat and radicle are extracted. The pixel distance between the farthest endpoint and the nearest intersection point on the radicle skeleton relative to the seed coat skeleton is calculated and converted into the actual distance, yielding the radicle length.

#### 2.2.2 Coarse segmentation

The Segment Anything Model (SAM) is a general segmentation framework trained on the SA-1B dataset, capable of zero-shot segmentation of unknown objects. Its architecture comprises an image encoder, a prompt encoder, and a lightweight mask decoder. The complete structural configuration is illustrated in Fig. 1. The image encoder is based on a Vision Transformer, pre-trained on ImageNet-21k and fine-tuned on the SA-1B dataset. The prompt encoder converts input prompts into high-dimensional representations, while the mask decoder efficiently generates segmentation masks by integrating image features with prompt information.

Due to the small size of the seed targets and the complex, diverse shapes of radicle, directly applying SAM to segment the entire seed image *I*_0_ yields suboptimal results. To address this, a YOLO-based seed detection model was trained in this study to locate seeds and generate prompt information, thereby improving the segmentation accuracy of SAM. The architecture of this detection model is shown in Fig. 1. As shown in Step 1 of Fig. 1, the YOLO detection model identifies bounding boxes for the seeds, which are used as prompts and input into SAM along with *I*_0_ to produce segmentation masks for the seeds.

**Fig. 2:**
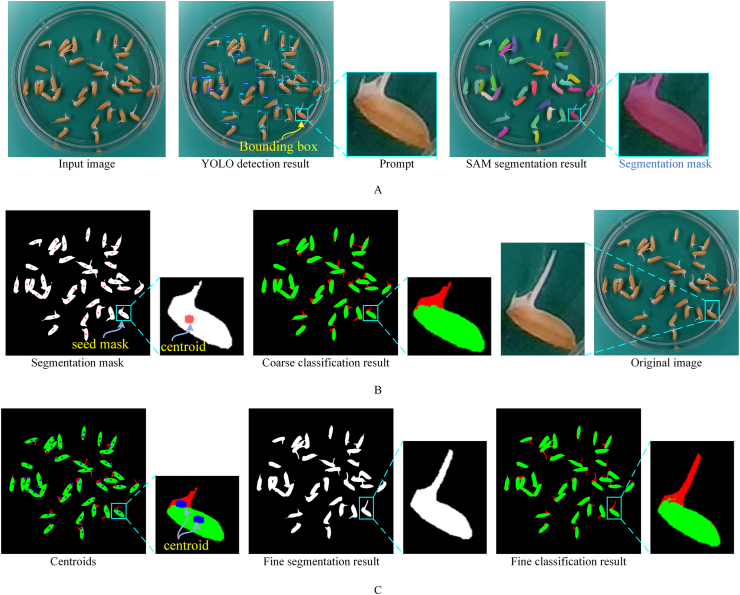
Multistage process of (A) coarse segmentation, (B) coarse classification, (C) fine segmentation and fine classification for rice seed images.

Fig. 2A illustrates examples of coarse segmentation for rice seed images. As shown in Fig. 2A, for some bounding boxes of germinated seeds, the boxes include the seed coat and part of the radicle, while another portion of the radicle lies outside the bounding box. The segmentation masks in Fig. 2A reveal that when the bounding box of each seed is used as a prompt for SAM, the segmentation results are constrained within the bounding box. This indicates that, due to the reliance on bounding box constraints during coarse segmentation, portions of the radicle may not be fully segmented.

#### 2.2.3 Coarse classification

Step 2 involves classifying each pixel in the coarse segmentation masks as either seed coat or radicle. This study designs a pixel classification algorithm based on a Gaussian model, constructing a Gaussian model for the seed coat using the color information of the centroids of the segmentation masks, and classifying pixels according to their probability values within the mask.

First, for each seed mask obtained in Step 1, the average values of all pixel x-coordinates and y-coordinates are calculated to determine the centroid position of the seed mask. As shown in Fig. 2B, the centroid of the seed mask is located at the seed coat position. This is primarily due to the significantly larger area of the seed coat compared to that of the radicle. Additionally, the bounding boxes obtained from YOLO detection only include the seed coat and a portion of the radicle that is close to the seed coat, neglecting the remaining part of the radicle.

Subsequently, we extract pixels within a 3 ​× ​3 area surrounding the centroid as training samples. The feature vector of each sample is defined as $x_{i}={\left(R-B,G-B\right)}^{T}$, where *R*, *G*, and *B* represent the red, green, and blue values of the corresponding pixels in the input image, respectively. Using these training samples, we establish a Gaussian model G=(m,C) for the seed coat, where *m* denotes the mean of the training samples, and *C* represents the covariance matrix of the training samples.

Once the Gaussian model is established, the probability *p*(*x*_*i*_) for the pixels belonging to the segmentation mask in the input image is calculated based on this model.

(1) $$p\left(x_{i}\right)=\exp\left[-0.5{\left(x_{i}-m\right)}^{T}C^{-1}\left(x_{i}-m\right)\right]$$

When *p*(*x*_*i*_) exceeds the threshold *T*, the pixel is classified as the seed coat; otherwise, it is considered as the radicle. Through extensive experiments, this study sets the threshold *T* to 0.006. The probability values for all pixels within the seed mask are calculated according to Eq. (1), thereby achieving the coarse classification of pixels. As shown in Fig. 2B, the Gaussian model accurately segments the seed mask into two parts: the seed coat and the radicle. However, for some seeds with longer radicles, a portion of the radicle may lie outside the bounding box during segmentation, leading to this part being misclassified as background.

#### 2.2.4 Fine segmentation and classification

Next, we proceed with fine segmentation and fine classification sequentially.

In Step 3, the fine segmentation method is similar to the coarse segmentation method, with the key difference being that the input to SAM includes centroid information. After coarse classification, each seed mask is divided into two parts: the seed coat and the radicle. We calculate the centroids for both parts and use the centroid coordinates of each seed mask as prompt points for input into SAM. Based on these prompts, SAM performs fine segmentation on the seed image, resulting in more precise segmentation masks.

In Step 4, the fine classification approach resembles the coarse classification method, but this step utilizes the centroids of the seed coat obtained from Step 3 as training samples. After establishing the Gaussian model for the seed coat, we calculate the probability *p*(*x*_*i*_) for each pixel in the seed mask obtained from fine segmentation according to Eq. (1).

Fig. 2C illustrates an example of fine segmentation and fine classification for a rice seed image. As shown in Fig. 2C, by inputting the centroids located at the seed coat and radicle into SAM, Step 3 achieves a complete segmentation of the radicle. This precise segmentation ensures that the radicle region obtained after classification in Step 4 is also intact.

#### 2.2.5 Radicle length

Through Step 4, we obtained the radicle mask. The next step is to measure the length of the radicle based on the radicle mask. To facilitate the explanation of the algorithm, we first provide the following definitions.

Definition 1 (Graph representation of skeleton)

The graph G=(V,E) is a representation of the skeleton *S*, where each pixel in S is considered a vertex in the vertex set V. An edge is established between two vertices if their corresponding pixels are connected within the 8-neighborhood. The edge weight is determined by the geometric relationship between the pixels: horizontally or vertically connected pixels have a weight of 1, while diagonally connected pixels have a weight of $\sqrt{2}$.

Definition 2 (Endpoint and intersection point)

In the graph G=(V,E) of skeleton *S*, an endpoint p_e_ is a vertex with degree 1, and an intersection point p_i_ is a vertex with degree 3 or more.

Definition 3 (Distance between a vertex and a graph)

The distance between a vertex $v_{1}\in V_{1}$ and a graph $G_{2}=\left(V_{2},E_{2}\right)$ is defined as:

(2) $$\text{dist}\left(v_{1},G_{2}\right)=\min_{v_{2}\in V_{2}}\text{dist}\left(v_{1},v_{2}\right)$$

where $\text{dist}\left(v_{1},v_{2}\right)$ is Euclidean distance between vertices *v*_1_ and *v*_2_.

Definition 4 (Geodesic distance between two vertices in a graph)

The geodesic distance between two vertices u,v∈V in a graph G=(V,E) is defined as the sum of the edge weights along the shortest path connecting u and v.

Algorithm 1 provides the method for calculating the pixel length of the radicle.

Algorithm 1 Calculation of radicle pixel length

**Table:** 

**Input:** Seed coat mask M_s_ and radicle mask M_r_ for a single seed.
1 Extract the skeletons S_s_ and S_r_ from M_s_ and *M*_*r*_, respectively.
2 Construct the graph representations G_s_ and G_r_ for S_s_ and S_r_ based on Definition 1.
3 Identify the endpoint p_e_ on G_r_ that is farthest from G_s_ according to Definition 2 and Definition 3.
4 Identify the intersection point p_i_ on G_r_ that is closest to G_s_ according to Definition 2 and Definition 3. 5 Compute the geodesic distance l_p_ between p_e_ and p_i_ on G_r_ based on Definition 4.
**Output:** Pixel length of the radicle l_p_.

**Fig. 3:**
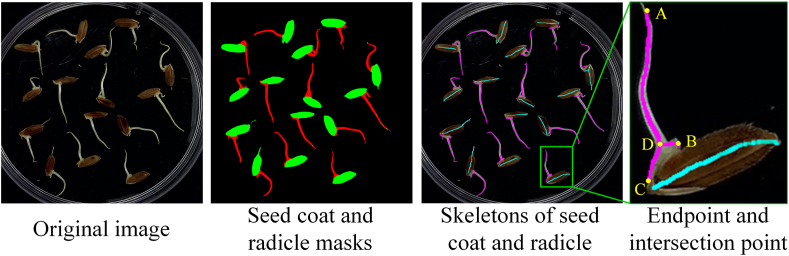
Visualization of skeleton, endpoint, and intersection point.

Below, we provide further elaboration on Algorithm 1. As illustrated in Fig. 3, we can utilize the Zhang-Suen Thinning Algorithm [38] to skeletonize the seed coat and radicle masks and construct the corresponding graphs. Fig. 3 presents a local magnification that includes three endpoints, A, B, and C, as well as an intersection point D. Endpoint A is the farthest from the skeleton, thus it is designated as *p*_*e*_. Point D serves as the intersection point *p*_*i*_. Finally, the geodesic distance between points A and D is taken as the pixel length of the radicle.

#### 2.2.6 Estimation of the true length of the radicle

Below, we present the estimation of the true length of the radicle.

Given that the seeds are classified as small targets, a close distance is required during imaging. Under such conditions, it is not feasible to use a full A4 sheet as a calibration reference. Therefore, in this study, an A4 sheet is cut to one sixty-fourth of its original size to serve as the calibration object. Assuming the camera position remains fixed during imaging, capturing a single image that includes the calibration object allows us to convert the pixel length of the radicle into its true length.

As shown in Fig. S1, we placed a one sixty-fourth-sized A4 sheet on a non-reflective black cloth for imaging. After capturing the image of the calibration object, we first applied morphological operations, including erosion and dilation, as well as Canny edge detection to extract the edge information. Subsequently, we selected the largest closed contour as the outline of the calibration object and identified the four points A′, B′, C′, and D′, which are closest to the vertices A, B, C, and D in the image, as the vertices of the calibration object. Finally, we calculated the pixel area of the calibration object using the discrete Green's theorem.

(3) $$S_{p}=\frac{1}{2}\left|\sum_{i=0}^{4}\left(x_{i}y_{i+1}-y_{i}x_{i+1}\right)\right|$$

where $\left(x_{i},y_{i}\right)$ represents the coordinates of any vertex of the calibration object, while $\left(x_{0},y_{0}\right)$ and $\left(x_{n},y_{n}\right)$ refer to the same vertex.

Furthermore, we can obtain the ratio *R* between the pixel length and the tree length:

(4) $$R=\frac{l_{p}}{l_{r}}=\sqrt{\frac{S_{p}}{S_{r}}}$$

where *S*_*r*_ is the true area of the one sixty-fourth-sized A4 sheet, and *l*_*p*_ and *l*_*r*_ represent the pixel length and the true length of the radicle, respectively. Once *R* is obtained, the true length of the radicle *l*_*r*_ can be determined as follows:

(5) $$l_{r}=\frac{l_{p}}{R}$$

#### 2.2.7 Experimental environment and execution settings

The experimental platform included a computer with an Intel® Core™ i9-10980 CPU and an NVIDIA GeForce RTX 4090 GPU. The system has 64 ​GB of internal memory, while the GPU is equipped with 24 ​GB of GDDR6X video memory. The software environment consists of Python 3.12 and PyTorch 2.3.

We first conducted seed segmentation experiments using the proposed algorithm and two comparative algorithms on the Riceseed1 dataset. Additionally, we performed segmentation ablation experiments. Following that, we measured the true length of the radicle on the Riceseed2 dataset. To validate the applicability of the proposed algorithm across different species, we conducted measurements of radicle pixel length on the Otherseed dataset, which includes maize, pearl millet, and rye. The code can be found online. The detailed training configurations for YOLOv8, UNet, and Mask-RCNN are summarized in Table 1.

**Table 1:** Training configurations for YOLOv8, UNet, and Mask-RCNN.

Parameter	YOLOv8	UNet	Mask-RCNN
Learning rate	0.01	1e-05	1e-04
Optimizer	SGD	RMSprop	Adam
Batch size	16	4	1
Epochs	300	300	300
Pre-training	No	No	No
LR scheduler	Cosine	ReduceLROnPlateau (monitor ​= ​'max', patience ​= ​5)	Step-LR (step ​= ​4, gamma ​= ​0.1)
Dataset	Riceseed2	Riceseed1	Riceseed1
Input size	Not specified	Not specified	Not specified

#### 2.2.8 Definition of segmentation evaluation metrics

In image segmentation tasks, commonly used evaluation metrics include the Dice coefficient (Dice), intersection over union (IoU), pixel accuracy (PA), and false positive rate (FPR).

Dice measures the consistency between the predicted region and the ground truth region.

(6) $$Dice=\frac{2\times \left|M_{p}\cap M_{g}\right|}{\left|M_{p}\right|+\left|M_{g}\right|}$$

where the predicted region *M*_*p*_ specifically denotes the combined area of seed coat and radicle, and *M*_*g*_ represents the ground truth region.

IoU measures the overlap between the predicted region and the ground truth region.

(7) $$IoU=\frac{\left|M_{p}\cap M_{g}\right|}{\left|M_{p}\cup M_{g}\right|}$$

In this context, the predicted region refers to the combined area of the seed body and radicle, which are treated as a single unified segmentation target.

PA evaluates the accuracy of the predicted segmentation region at the pixel level.

(8) $$PA=\frac{\left|M_{p}\cap M_{g}\right|}{\left|M_{g}\right|}$$

where $\left|M_{p}\cap M_{g}\right|$ denotes the number of correctly predicted pixels, and $\left|M_{g}\right|$ denotes the total number of pixels in the ground truth region.

FPR measures the proportion of pixels misclassified as part of the segmentation region.

(9) $$FPR=\frac{\left|M_{p}\setminus M_{g}\right|}{\left|M_{bg}\right|}$$

where $M_{p}\setminus M_{g}$ represents the background pixels incorrectly classified as part of the segmentation region, and $M_{bg}$ denotes the total number of pixels not belonging to the ground truth segmentation region.

To address component-specific segmentation quality, three extension metrics were implemented. The seed coat IoU (IoU_sc_) assesses seed body segmentation accuracy by computing the intersection-over-union ratio of seed coat regions. Similarly, the radicle IoU (IoU_rd_) quantifies accuracy for the germinated root structure using analogous computation. Background discrimination performance was measured by IoU_bg_, defined as the overlap ratio for background regions. These component metrics collectively inform the mean IoU (mIoU), derived by averaging IoU_bg_, IoU_sc_, and IoU_rd_.

(10) $$IoU_{sc}=\frac{\left|M_{p}^{sc}\cap M_{g}^{sc}\right|}{\left|M_{p}^{sc}\cup M_{g}^{sc}\right|}$$

(11) $$IoU_{rd}=\frac{\left|M_{p}^{rd}\cap M_{g}^{rd}\right|}{\left|M_{p}^{rd}\cup M_{g}^{rd}\right|}$$

(12) $$IoU_{bg}=\frac{\left|M_{p}^{bg}\cap M_{g}^{bg}\right|}{\left|M_{p}^{bg}\cup M_{g}^{bg}\right|}$$

(13) $$mIoU=\frac{1}{3}\left(IoU_{bg}+IoU_{sc}+IoU_{rd}\right)$$

## 3 Results

### 3.1 Segmentation performance of LenRuler

**Fig. 4:**
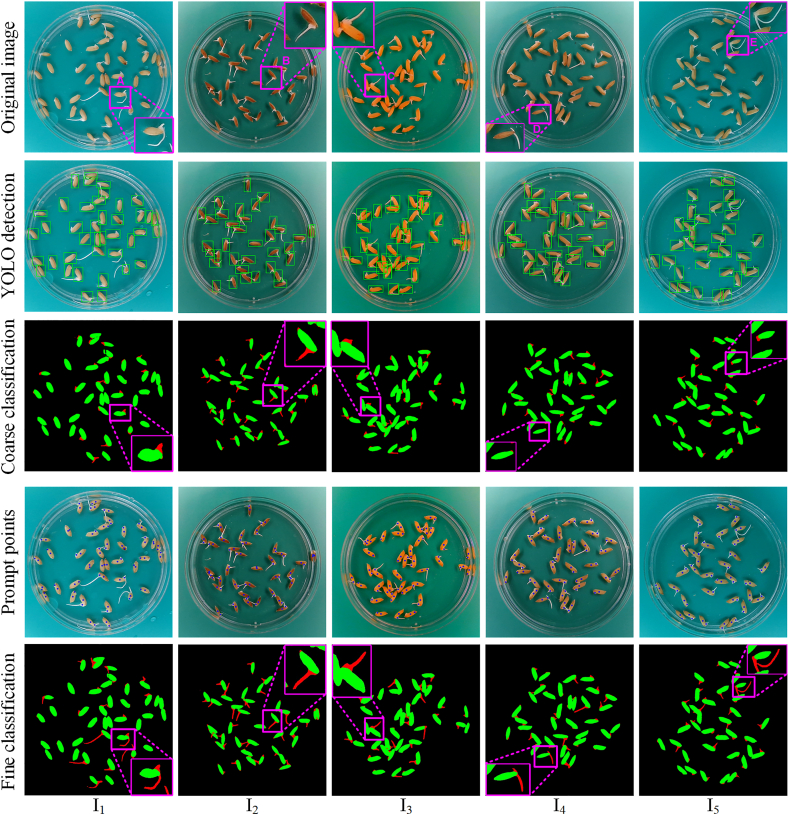
The segmentation results of five rice seed images obtained using LenRuler.

This subsection provides a comprehensive analysis of the segmentation performance of LenRuler. Five rice seed images were selected from the Riceseed1 dataset, which vary in seed coat color, background color, seed distribution density, radicle length, and lighting condition. Fig. 4 illustrates the classification results of these five images using LenRuler. Since classification can more clearly differentiate between the seed coat and radicle, and it is conducted as a binary classification based on the segmentation results, Fig. 4 uses the classification results to illustrate the segmentation performance. A comparison between the coarse classification and fine classification results reveals that the segmentation performance of SAM is significantly enhanced when centroids derived from the coarse classification results are used as prompt points.

For example, in image I_1_, seed A has a relatively long radicle. The bounding box detected by YOLO includes the seed coat along with a small portion of the radicle. When this bounding box is input into SAM, the coarse classification result obtained through the Gaussian model shows that the radicle mask includes only a small segment of the radicle. However, after providing SAM with additional prompt points, the fine classification result produces a radicle mask that successfully captures the entire radicle. Similarly, for seeds B, C, and D in the other four images, LenRuler accurately extracts the complete radicles. In image I_5_, where seed E has both a germinated plumule and radicle, LenRuler effectively identifies and extracts both structures.

To evaluate the generalization ability of LenRuler under challenging imaging conditions, we further tested it on a set of seed images exhibiting various complex scenarios, including awns, shadows, seed adhesion, water bubbles, ghosting artifacts on the Petri dish, and red background. As shown in Fig. S2, LenRuler maintains accurate segmentation performance across all these conditions. Specifically, it successfully distinguishes tightly adhered seeds, correctly identifies radicles in the presence of shadows or background reflections, and remains robust against image distortions and color variations.

### 3.2 Ablation experiment

To evaluate the contribution of each component in the segmentation pipeline (Steps 1–3 of LenRuler), we conducted an ablation study using five different model variants. These ablations help isolate the effect of coarse segmentation, coarse classification, and fine segmentation. Notably, Steps 4 (fine classification) and 5 (radicle length measurement) were excluded from this analysis as they depend on the segmentation results.

Method definitions are as follows (also shown in Table 2):

(i) Method 1 (Full Image ​+ ​SAM only): Applies SAM directly to the full image without any prompt or classification.
(ii) Method 2 (Mask-RCNN): Replaces the SAM-based segmentation with Mask-RCNN.
(iii) Method 3 (YOLO ​+ ​SAM): Uses YOLO bounding boxes as prompts for SAM, without classification.
(iv) Method 4 (YOLO ​+ ​SAM ​+ ​Seed coat centroid): Adds seed coat centroids as prompt points.
(v) Method 5 (YOLO ​+ ​SAM ​+ ​Seed coat ​+ ​Radicle centroids): Full LenRuler pipeline, including both seed coat and radicle centroids as prompts.

**Table 2:** Configuration of five segmentation ablation methods.

Method	Step 1: Coarse Segmentation	Step 2: Coarse Classification	Step 3: Fine Segmentation
Method1	SAM applied to full image	×	×
Method2	Mask-RCNN	✓	✓
Method3	✓	×	×
Method4	✓	✓	Seed coat centroids only
Method5	✓	✓	✓

To further validate the threshold value *T* ​= ​0.006 used in Method5 for Gaussian-based classification, we conducted a sensitivity analysis on the same 50 images as in the ablation study to confirm the appropriateness of the threshold *T* ​= ​0.006. Specifically, the threshold *T* was varied from 0.001 to 0.010 in increments of 0.001. For each threshold value, segmentation performance was evaluated using three metrics: Dice, IoU, and PA. The FPR metric was excluded from the analysis due to its relatively small values, which makes it less suitable for visualization. The results are presented in Fig. S3. As shown in Fig. S3, all three metrics reach their peak values when *T* ​= ​0.006, indicating that this threshold yields the best overall segmentation performance under the current experimental settings. Therefore, *T* ​= ​0.006 was adopted as the optimal threshold in this study.

To ensure a fair comparison, we randomly selected 50 test images from Riceseed1. Fig. S4 provides visual comparisons, and Table 3 reports the quantitative metrics.

**Table 3:** The metric values of the five algorithms on 50 images.

	Dice↑	IoU↑	PA↑	FPR↓	IoU_sc_↑	IoU_rd_↑	mIoU↑
Method1	0.778 ​± ​0.226	0.683 ​± ​0.251	0.737 ​± ​0.280	0.029 ​± ​0.015	0.621 ​± ​0.240	0.580 ​± ​0.263	0.628 ​± ​0.229
Method2	0.725 ​± ​0.118	0.582 ​± ​0.139	0.852 ​± ​0.062	0.096 ​± ​0.104	0.540 ​± ​0.133	0.493 ​± ​0.148	0.605 ​± ​0.131
Method3	0.943 ​± ​0.025	0.847 ​± ​0.057	0.915 ​± ​0.046	**0.010** ​± ​**0.004**	0.861 ​± ​0.044	0.829 ​± ​0.069	0.879 ​± ​0.048
Method4	0.916 ​± ​0.035	**0.913** ​± ​**0.018**	0.937 ​± ​0.066	0.044 ​± ​0.013	**0.930** ​± ​**0.024**	0.889 ​± ​0.031	0.918 ​± ​0.026
Method5	**0.955** ​± ​**0.010**	0.894 ​± ​0.044	**0.944** ​± ​**0.021**	0.014 ​± ​0.006	0.912 ​± ​0.018	**0.901** ​± ​**0.027**	**0.936** ​± ​**0.022**

As shown in Fig. S4, Method 1 produces under-segmented results, especially when seeds are in contact (e.g., I2 and I5). Method 2 suffers from both over- and under-segmentation, misclassifying background pixels as seeds. Method 3 improves accuracy by incorporating bounding box prompts but still lacks structural detail. Method 4, with seed coat centroids, improves object boundaries slightly. Method 5, the full LenRuler pipeline, achieves the most complete and accurate segmentation.

Compared to Method 4, Method 5 improves IoU_rd_ by 1.35 ​% and overall mIoU by 1.96 ​%, indicating the importance of including both centroid types. Likewise, Dice improves by 1.27 ​% over Method 3, further validating the benefit of classification-guided prompts.

### 3.3 Comparison of different segmentation algorithms

This section presents a detailed performance comparison between the proposed method and two widely used segmentation algorithms, UNet and Mask-RCNN. As described in Section 2.1, both UNet and Mask-RCNN were trained using the training set of the Riceseed1 dataset. The proposed method follows the configuration of Method 4 introduced in the ablation experiments in Section 3.2.

**Fig. 5:**
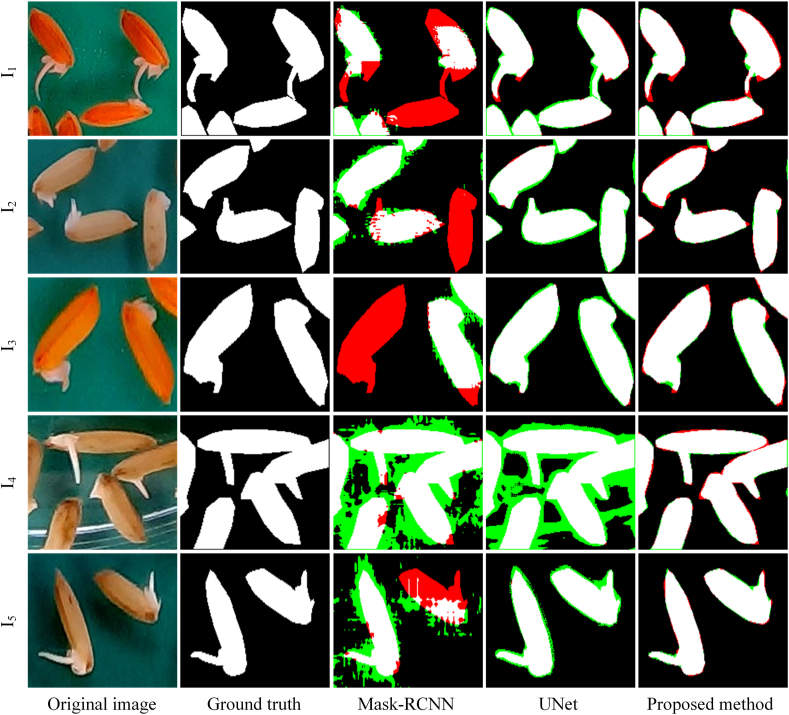
The local segmentation results of three algorithms on five images. White: correctly segmented seed/radicle; red: missed regions; green: false-positive background.

To ensure a fair comparison, the segmentation performance of all three methods was evaluated on the same set of 50 rice seed images. These images are the same as those used in the ablation experiments in Section 3.2, which were randomly selected from the test set of the Riceseed1 dataset. Fig. 5 illustrates local segmentation results for five representative images, where white regions indicate correctly segmented targets, red regions highlight missed target regions, and green regions represent non-target regions mistakenly identified as targets.

Mask-RCNN shows both oversegmentation and undersegmentation across all images. It often misclassifies background areas as seed regions, especially near the image borders, revealing its weakness in handling small, thin structures like radicles under cluttered backgrounds. This is likely due to its reliance on anchor-based proposals, which lack precision for such fine targets.

UNet performs better in locating seed regions but frequently produces ambiguous boundaries. As seen in image I_4_, it wrongly includes parts of the Petri dish, and in dense areas, it fails to separate overlapping seeds, resulting in merged masks. This is largely caused by its lack of object-level understanding.

In contrast, the proposed method produces cleaner, more accurate masks by combining bounding boxes and centroids as prompts into SAM. The Gaussian-based classification further improves radicle boundary refinement. In complex scenes, such as image I_4_, it consistently delivers complete and precise segmentation, attributed to its coarse-to-fine, prompt-driven design.

**Fig. 6:**
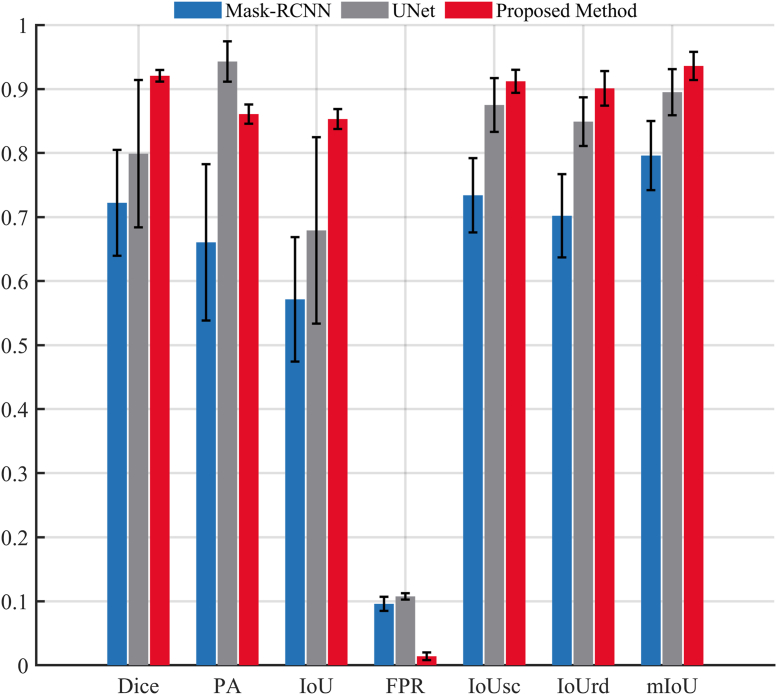
Performance evaluation of three algorithms. Black error bars: standard deviation.

Fig. 6 compares the performance of the three algorithms in terms of the Dice, PA, IoU, FPR, IoU_sc_, IoU_rd_, and mIoU metrics. As shown in Fig. 6, the proposed method outperforms both UNet and Mask-RCNN across all major metrics. It achieves the highest Dice coefficient (0.921), IoU (0.853), and mIoU (0.936), indicating superior overall segmentation accuracy. For radicle segmentation, it also records the best IoU_rd_ (0.901) and the lowest false positive rate (FPR ​= ​0.014), demonstrating strong capability in extracting fine structures with minimal background confusion.

UNet attains the highest pixel accuracy (PA ​= ​0.943) but lags behind in Dice (0.799), IoU (0.679), and IoU_rd_ (0.849), suggesting that although it correctly identifies most foreground pixels, it struggles with precise boundary delineation. Mask-RCNN performs worst across all metrics, with Dice ​= ​0.722, IoU ​= ​0.571, and FPR ​= ​0.096, reflecting frequent oversegmentation and background misclassification.

In terms of consistency, the proposed method presents the lowest standard deviations in Dice (±0.009), IoU (±0.016), and mIoU (±0.022), confirming its robustness across diverse image conditions. In contrast, UNet and Mask-RCNN exhibit much higher variances, particularly in IoU (±0.146 for UNet, ±0.097 for Mask-RCNN), indicating unstable performance.

Overall, these results validate the effectiveness of the proposed prompt-guided, two-stage segmentation framework. It not only improves accuracy in both seed coat and radicle regions but also provides stable results under varying image complexities.

### 3.4 Radicle length measurement for rice seeds

**Fig. 7:**
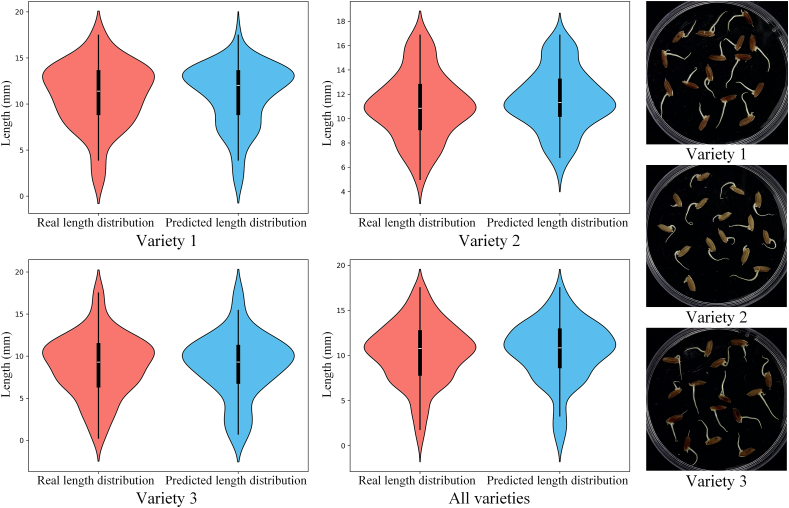
Comparison of predicted and manually measured radicle length distributions across three rice varieties. Violin plots illustrate the distributions of real (red) and predicted (blue) radicle lengths for each variety and for all samples combined, based on 120 test images from the Riceseed2 dataset. Representative images for each variety are shown on the right. The close alignment between predicted and ground truth values demonstrates the accuracy and robustness of LenRuler across genetically diverse phenotypes.

To assess the accuracy of LenRuler in measuring radicle length, we conducted an evaluation using 120 images from the Riceseed2 test set. The radicle length of each germinated seed was measured and compared with manually annotated ground truth values. Fig. 7 presents a comparative analysis of the distribution between the predicted radicle lengths and the manual measurements, alongside representative images from three rice varieties. As depicted in Fig. 7, the predicted radicle lengths closely align with the ground truth across all three rice varieties, further demonstrating the robustness and accuracy of the proposed method.

**Fig. 8:**
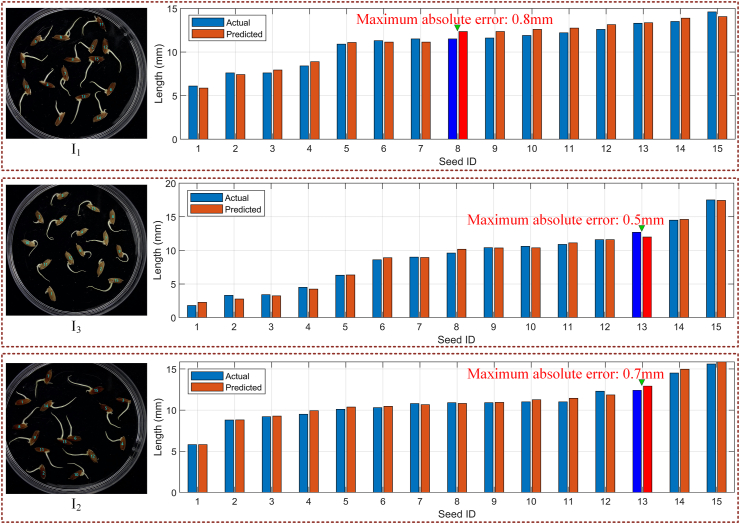
Comparison of predicted and true radicle lengths in three rice images.

We selected one image from each of the three rice varieties and visualized the radicle length predictions and ground truth values using bar charts. The numbers below each bar in the chart correspond to the seed indices labeled in the image on the left. As shown in Fig. 8, the predicted radicle lengths closely match the ground truth values, further demonstrating the effectiveness of the proposed algorithm. In I_1_, the absolute error of Seed 8 is the largest, reaching 0.8 ​mm, which is also the maximum absolute error across all measured seeds. To provide a comprehensive quantitative evaluation on the full test set, we calculated standard statistical indicators across all 120 images: the MAE was 0.273 ​mm and the R^2^ was 0.982. These metrics confirm the high accuracy of LenRuler across the entire dataset. Additionally, we randomly selected 10 images from the 120 images and plotted the comparison between manual measurements and LenRuler predictions for each seed in these images. As shown in Fig. S5, most data points lie close to the reference line, indicating that the predicted values are highly consistent with the true values and minimizing overall error.

### 3.5 Validation of radicle length measurement in other crops

**Fig. 9:**
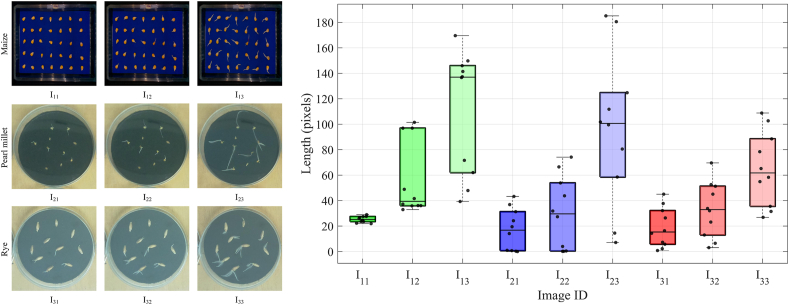
Predicted results of radicle length for three crops. Maize (green), pearl millet (blue), and rye (orange), with linear trend lines fitted to median values. Left: representative seed images from each group (I_11_–I_33_).

This section validates the performance of the proposed algorithm to other crops. The experiment utilized three crops from the Otherseed dataset: maize, pearl millet, and rye. Due to the absence of reference markers in the dataset, true radicle lengths could not be measured. Instead, radicle lengths were measured in pixel units. To facilitate validation, three images were selected for each crop, representing seeds with short, medium, and long radicles. Fig. 9 presents the predicted radicle length distributions for these nine images.

As shown in the boxplots for maize in Fig. 9, radicle lengths in images I_11_, I_12_, and I_13_ display an increasing trend, consistent with visual observations. For pearl millet, the radicle length in I_23_ is notably greater than that in I_21_ and I_22_, a difference clearly reflected in the corresponding boxplots in Fig. 9, where I_23_ is significantly higher than I_21_ and I_22_. For rye, most seeds in I_32_ and I_33_ simultaneously developed both radicles and plumules. The proposed algorithm determines radicle length by identifying the endpoint farthest from the seed coat, effectively minimizing interference from plumules. Typically, radicle length exceeds plumule length, ensuring the results remain unaffected by the presence of plumules. The boxplots for I_31_, I_32_, and I_33_ in Fig. 9 are consistent with the observed radicle length distributions, also showing an increasing trend.

## 4 Discussion

In this study, we propose LenRuler, an automated method for measuring radicle length with a rice-centric design and multicrop validation. The system integrates SAM, YOLO detection, and a Gaussian-based classification strategy to achieve high-precision segmentation and accurate length estimation of radicles. Based on our experiments and comparisons, LenRuler demonstrates substantial advantages in segmentation accuracy, measurement precision, and cross-species applicability.

### 4.1 Key contributions and innovations

The most significant innovation of LenRuler lies in its coarse-to-fine segmentation pipeline, where bounding boxes and centroids are iteratively used as prompts for SAM. This strategy combines the flexibility of prompt-based segmentation with a classification-guided refinement mechanism, resulting in high-quality seed coat and radicle masks. Moreover, we introduced a geodesic-based length computation algorithm, which accurately calculates radicle length even in the presence of curvature—something that traditional Euclidean-based methods or simple contour length approximations often fail to address.

Another key strength is the low annotation burden. Unlike conventional segmentation-based methods that require large-scale pixel-level annotations, LenRuler only needs bounding boxes for training the detection model. This makes it much easier to extend the method to new crops.

### 4.2 Performance and comparison

Through extensive experiments on rice, maize, pearl millet, and rye datasets, LenRuler achieved superior segmentation accuracy compared to widely used models such as UNet and Mask-RCNN. The Dice coefficient reached 0.955, and pixel accuracy exceeded 94 ​%, indicating the robustness of the segmentation pipeline. The two-stage segmentation approach not only reduces the omission of radicle regions but also significantly improves segmentation precision. Quantitative evaluation on the Riceseed2 test set (120 images) demonstrated high measurement accuracy, with MSE ​= ​0.121 ​mm and R^2^ ​= ​0.982, which exceeds typical requirements for germination and seed vigor studies.

Furthermore, our ablation studies confirmed that incorporating centroids of both seed coat and radicle significantly improves segmentation outcomes, validating the necessity of multi-stage prompt engineering in the SAM framework.

### 4.3 Comparison with traditional methods

Traditional tools like ImageJ and GERMINATOR rely heavily on manual operations, which are time-consuming and error-prone. Even recent deep learning models such as YOLO-r and SeedQuant focus primarily on germination status detection rather than accurate length measurement. Although methods like YOLOv8-segANDcal attempt to estimate radicle length, they still require full seed mask annotations and lack generalizability. In contrast, LenRuler offers a fully automated, annotation-efficient, and extensible solution to radicle length measurement, with demonstrated accuracy and flexibility.

### 4.4 Limitations and future work

While LenRuler performs well under controlled conditions, several limitations remain. The current dataset was collected in a laboratory setting with clean backgrounds, uniform lighting, and minimal seed overlap, which simplifies segmentation tasks and does not fully represent the variability and complexity of real-world environments. In particular, the method's generalization capacity under varying lighting and background conditions has not been systematically evaluated, limiting the strength of claims regarding its broad applicability. Specifically, the dataset lacks instances of large-scale overlapping or coinciding radicles, which are common in high-throughput or field scenarios. This relative simplicity means that the distinction between individual seeds and radicles is clear, and the overall complexity is low, making the current pipeline less robust under more stringent sample collection requirements in practical applications.

Challenges such as overlapping radicles, blurred boundaries, inconsistent image quality, and high-density seed distributions could adversely affect segmentation accuracy and robustness. Additionally, although the YOLO-based detection module is efficient, it requires retraining for different crop species, which limits its generalizability across diverse datasets. Moreover, training the YOLO model relies on a sufficiently large and consistently annotated dataset. Since the target objects—seeds and radicles—are small and often overlapping, manual annotation of bounding boxes is labor-intensive and time-consuming. The training process itself is computationally expensive, especially when optimizing for high-resolution and small-object detection, which increases the deployment barrier in resource-constrained environments.

In future work, we aim to enhance LenRuler by:

1. Incorporating more diverse and challenging datasets, including those with higher degrees of radicle overlap and complex seed distributions, to better simulate real-world scenarios.
2. Conducting systematic multi-environment evaluations under varying lighting conditions, background textures, and imaging setups to rigorously assess model generalization and robustness.
3. Introducing semi-supervised learning or active learning strategies to reduce annotation costs during YOLO training, and exploring lightweight or pre-trained detection backbones to lower computational overhead.
4. Improving cross-species adaptability through foundation models or domain adaptation techniques, reducing the need for retraining across different crops.
5. Expanding phenotyping capabilities to include traits such as plumule detection, root system analysis, and dynamic growth tracking, enabling more comprehensive plant phenotype assessments.
6. Developing guidelines for optimizing sample collection procedures to balance practical constraints and the need for robust segmentation in challenging environments.
7. Introducing adaptive thresholding strategies and incorporating spatial or texture-aware features to improve segmentation robustness under diverse imaging conditions.

### 4.5 Broader applicability

The core idea of LenRuler—using promptable segmentation combined with structural analysis—can be readily extended beyond seed phenotyping. For instance, tasks such as root phenotyping, shoot length measurement, and fruit shape analysis could benefit from similar strategies. In the context of radicle measurement, our results demonstrate the robustness and accuracy of LenRuler across different species and growth stages. These strengths suggest that LenRuler holds significant potential for integration into high-throughput breeding pipelines and smart agriculture systems, where automated, precise, and scalable phenotyping tools are increasingly essential.

In summary, LenRuler provides a robust, accurate, and extensible framework for radicle length measurement in rice and other validated crops, significantly advancing the automation and precision of seed phenotyping.

## 5 Conclusions

In this study, we proposed LenRuler, an automated and accurate method for radicle length measurement, specifically optimized for rice seeds and validated across multiple crop species. The method integrates SAM-based segmentation, YOLO-based detection, and Gaussian-based classification within a coarse-to-fine segmentation pipeline, enabling precise extraction of seed coat and radicle regions and accurate geodesic-based length computation.

On the rice-specific Riceseed1 dataset, LenRuler achieved a Dice coefficient of 0.955 and pixel accuracy of 0.944, demonstrating excellent segmentation performance. In the Riceseed2 test set, the MAE was 0.273 ​mm and the R^2^ was 0.982, confirming the method's high accuracy and robustness under practical conditions.

To assess its adaptability, LenRuler was further tested on the Otherseed dataset, which includes maize (*Zea mays*), pearl millet (*Pennisetum glaucum*), and rye (*Secale cereale*). The predicted radicle lengths for these species were consistent with observed growth patterns, indicating that the method can be extended to other crops with validated generalizability.

Compared to traditional manual or fully supervised approaches, LenRuler significantly reduces annotation effort by using bounding boxes and seed centroids as prompts, making it more efficient and scalable for high-throughput phenotyping tasks.

In summary, LenRuler provides a reliable and automated solution for accurate radicle length measurement in rice, with its effectiveness also verified in other crop species. Future work will focus on expanding the framework to additional traits such as plumule length and root system architecture, and on improving adaptability through general-purpose detection models suitable for diverse agricultural scenarios.

## Author contributions

J. Z. and Z. H. developed the software and methodology and wrote the original draft. H. H., Q. N. and Y. P. curated the data. Y. M. and X. H. reviewed and edited the manuscript and provided resources. Y. M. and X. H. supervised the research.

## Funding

This work was supported by the Yuelushan Laboratory Breeding Project (YLS-2025-ZY02006) to X.H.

## Data availability

The code and software are available on GitHub at https://github.com/cccccabbage/LenRuler.

## Competing interests

The authors declare that they have no competing interests.

## References

[1] Zhao J. (2024). YOLOrot2. 0: a novel algorithm for high-precision rice seed size measurement with real-time processing. Smart Agric. Technol..

[2] Jin C. (2025). Application of deep learning for high-throughput phenotyping of seed: a review. Artif. Intell. Rev..

[3] Liu H., Xu Z. (2023).

[4] Qiao Y., AI (2022).

[5] Visakh R.L. (2024).

[6] Jia J. (2024). OsJMJ718, a histone demethylase gene, positively regulates seed germination in rice. Plant J..

[7] Yoshida H. (2022). Genome-wide association study identifies a gene responsible for temperature-dependent rice germination. Nat. Commun..

[8] Fu D. (2025). Molecular mechanisms of rice seed germination. New Crops.

[9] Xu M. (2024). OsSCYL2 is involved in regulating ABA signaling-mediated seed germination in rice. Plants.

[10] Zhao J. (2023). Deep-learning-based automatic evaluation of rice seed germination rate. J. Sci. Food Agric..

[11] Tobe K. (2000). Seed germination and radicle growth of a halophyte, Kalidium caspicum (Chenopodiaceae). Ann. Bot..

[12] Mangrich M.E. (2006). Radicle length and osmotic stress affect the chilling sensitivity of cucumber radicles. Crop Sci..

[13] Biswas N. (2017). Correlation between superoxide content and radicle length during seed germination in different types of Indian mustard. Seed Res..

[14] Schneider C.A. (2012). NIH image to ImageJ: 25 years of image analysis. Nat. Methods.

[15] Joosen R.V. (2010). GERMINATOR: a software package for high-throughput scoring and curve fitting of arabidopsis seed germination. Plant J..

[16] Mallu T.S. (2021). New pre-attachment striga resistant sorghum adapted to African agro-ecologies. Pest Manag. Sci..

[17] Jhaj S.K. (2013). The effect of salinity on hypocotyl and radicle length of Arabidopsis thaliana. Expedition.

[18] Hernández-Herrera R.M. (2023). Seaweed extract components are correlated with the seeds germination and growth of tomato seedlings. Seeds.

[19] Yao Q. (2024). SGR-YOLO: a method for detecting seed germination rate in wild rice. Front. Plant Sci..

[20] Braguy J. (2021). SeedQuant: a deep learning-based tool for assessing stimulant and inhibitor activity on root parasitic seeds. Plant Physiol..

[21] Peng Q. (2022). Automatic monitoring system for seed germination test based on deep learning. J. Electr. Comput. Eng..

[22] Thakur P.S. (2022). Deep transfer learning based photonics sensor for assessment of seed-quality. Comput. Electron. Agric..

[23] Wu Y. (2024). YOLOv8-segANDcal: segmentation, extraction, and calculation of soybean radicle features. Front. Plant Sci..

[24] Sohan M. (2024). International Conference on Data Intelligence and Cognitive Informatics.

[25] Khoroshevsky F. (2024). Automatic root length estimation from images acquired in situ without segmentation. Plant Phenomics.

[26] Pound M.P. (2017). AutoRoot: open-source software employing a novel image analysis approach to support fully-automated plant phenotyping. Plant Methods.

[27] Slovak R. (2014). A scalable open-source pipeline for large-scale root phenotyping of arabidopsis. Plant Cell.

[28] Betegón-Putze I. (2019). My ROOT: a method and software for the semiautomatic measurement of primary root length in arabidopsis seedlings. Plant J..

[29] Kimura K., Yamasaki S. (2001). Root length and diameter measurement using NIH image: application of the line-intercept principle for diameter estimation. Plant Soil.

[30] Li H. (2025). Swin-Unet++: a study on phenotypic parameter analysis of cabbage seedling roots. Plant Methods.

[31] González A. (2020). MyROOT 2.0: an automatic tool for high throughput and accurate primary root length measurement. Comput. Electron. Agric..

[32] Torres-Lomas E. (2024).

[33] Redmon J. (2016). Proceedings of the IEEE Conference on Computer Vision and Pattern Recognition.

[34] Ronneberger O. (2015). Medical Image Computing and computer-assisted intervention–MICCAI 2015: 18Th International Conference, Munich, Germany, October 5-9, 2015, Proceedings, Part III 18.

[35] Dollár K.H.G.G.P., Girshick R. (2017). Mask r-cnn. Proceedings of the IEEE international conference on computer vision.

[36] Colmer J. (2020). SeedGerm: a cost-effective phenotyping platform for automated seed imaging and machine-learning based phenotypic analysis of crop seed germination. New Phytol..

[37] Genze N. (2020). Accurate machine learning-based germination detection, prediction and quality assessment of three grain crops. Plant Methods.

[38] Zhang T.Y., Suen C.Y. (1984). A fast parallel algorithm for thinning digital patterns. Commun. ACM.

