# Supplementary material for: LenRuler: a rice-centric method for automated radicle length measurement with multicrop validation
Source: Plant Phenomics. 2025 Sep 8;7(3):100103. doi: 10.1016/j.plaphe.2025.100103 (PMC12710053; doi:10.1016/j.plaphe.2025.100103)
Supplement: Multimedia component 1 [file mmc1.docx]

Supplementary Materials

**Table S1.** Summary of datasets used in this study (Riceseed1, Riceseed2, Otherseed)

| Dataset | Varieties | Images | Seeds | Training set | Test set | Reference |
| --- | --- | --- | --- | --- | --- | --- |
| Riceseed1 | Rice^1^ | 1,211 | 44,660 | - | 50 | [Zhao, Ma, Yong, Zhu, Wang, Luo, Wei and Huang [5]](#_ENREF_5) |
| Riceseed2 | Rice | 600 | 10,800 | 480 | 120 | - |
|  | Maize^2^ | 115 | 4,025 | 92 | 23 | [Colmer, O'Neill, Wells, Bostrom, Reynolds, Websdale, Shiralagi, Lu, Lou and Le Cornu [16]](#_ENREF_16) |
| Otherseed | Pearl millet^3^ | 7954 | 79,540 | - | - | [Genze, Bharti, Grieb, Schultheiss and Grimm [17]](#_ENREF_17) |
|  | Rye^3^ | 7695 | 923,400 | - | - | [Genze, Bharti, Grieb, Schultheiss and Grimm [17]](#_ENREF_17) |

^1^ <https://www.kaggle.com/jinfengzhao/riceseedgermination>

^2^ <https://github.com/Crop-Phenomics-Group/SeedGerm/releases>

^3^ <https://data.mendeley.com/datasets/4wkt6thgp6/2>


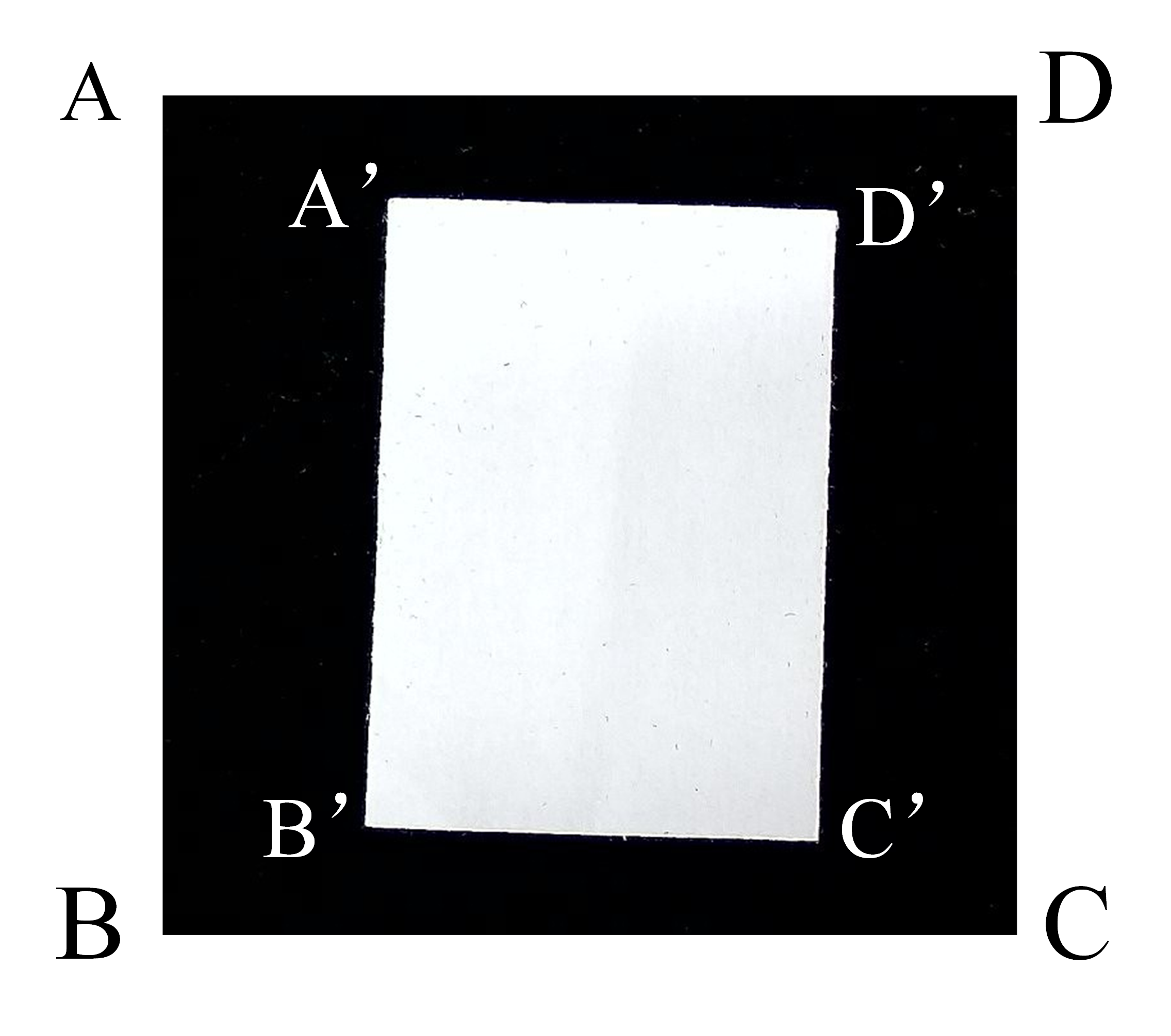


**Fig. S1.** Calibration reference object.

| Original image | Segmentation results |
| --- | --- |
| 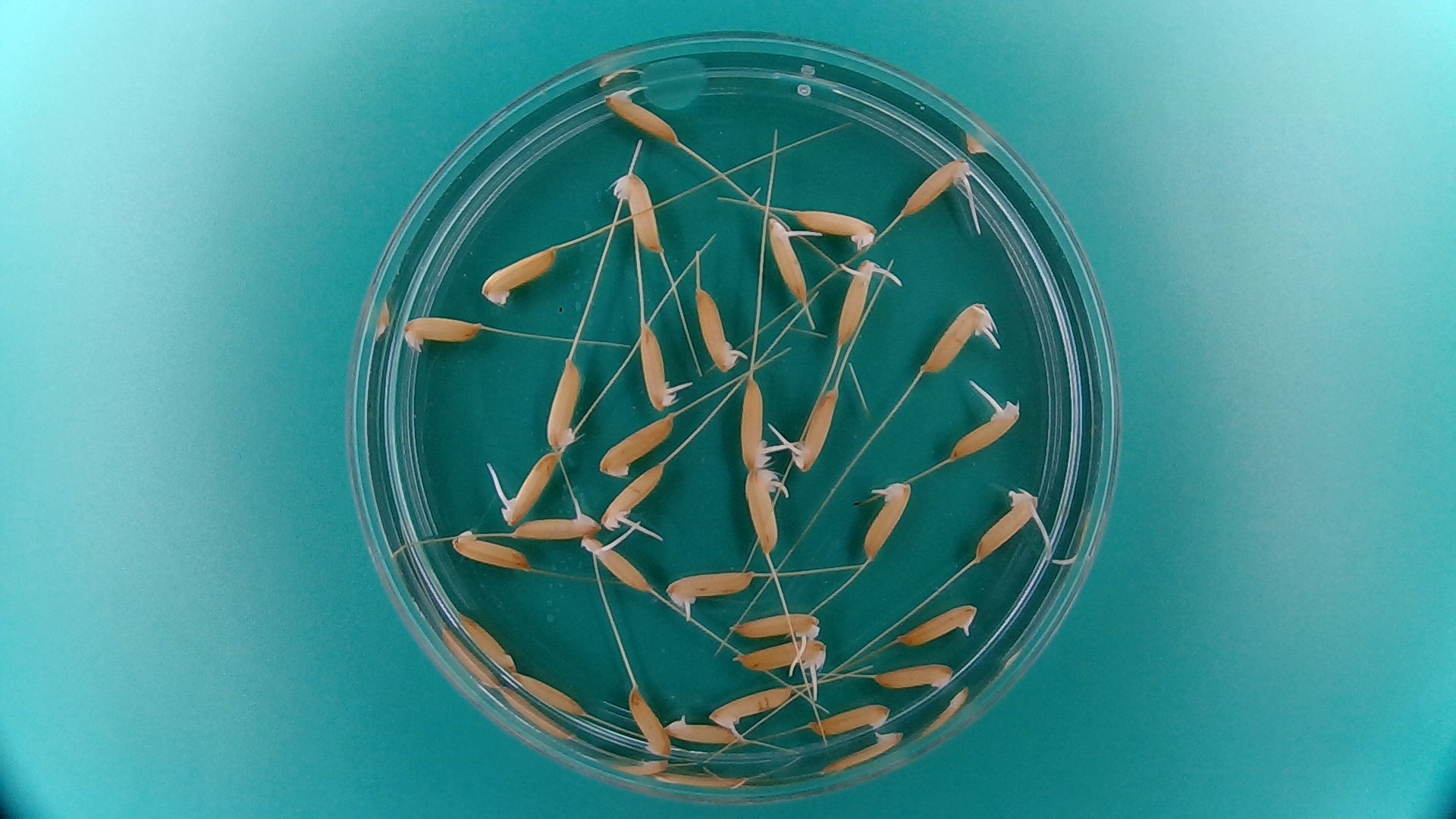 | 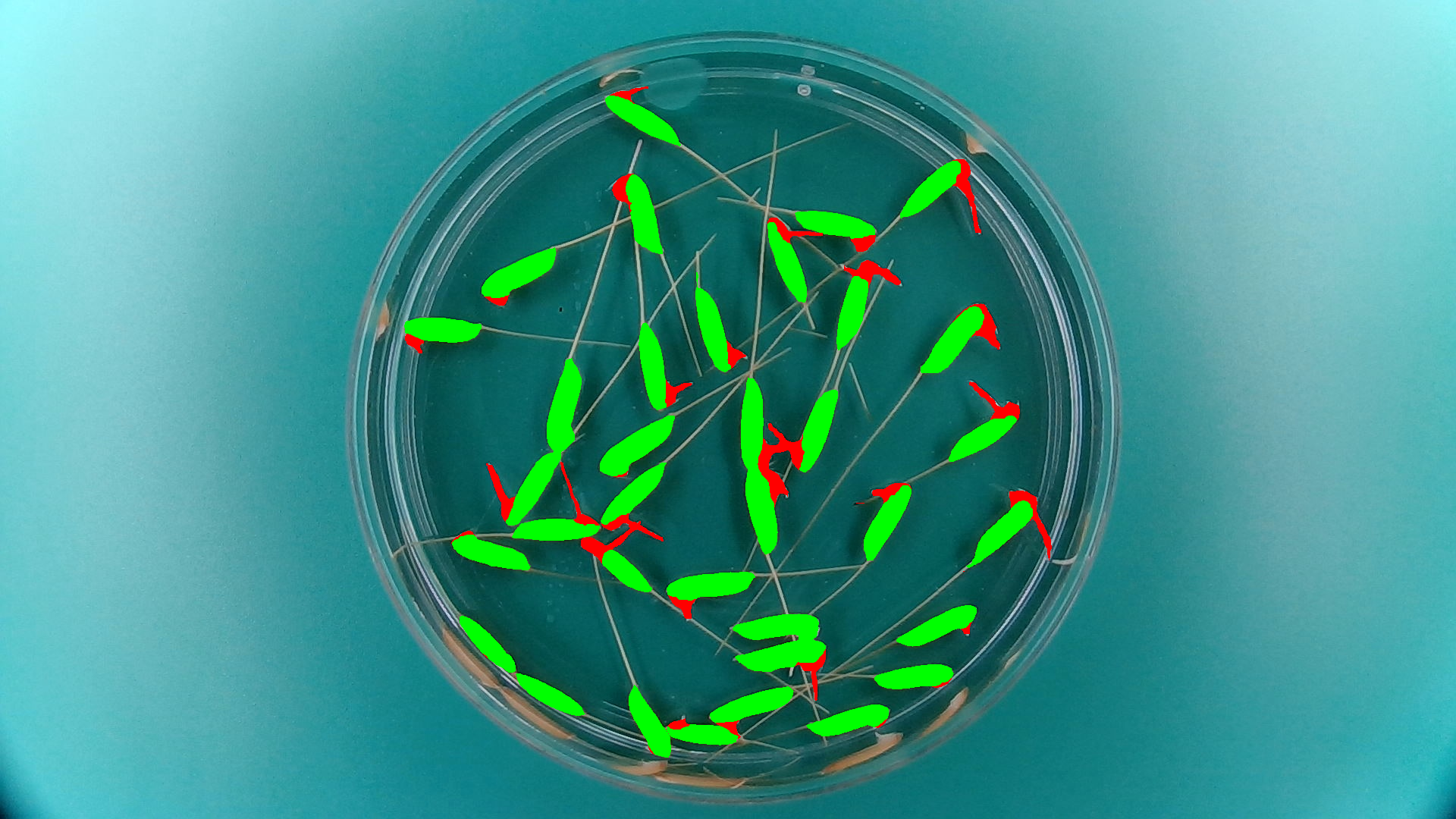 |
| 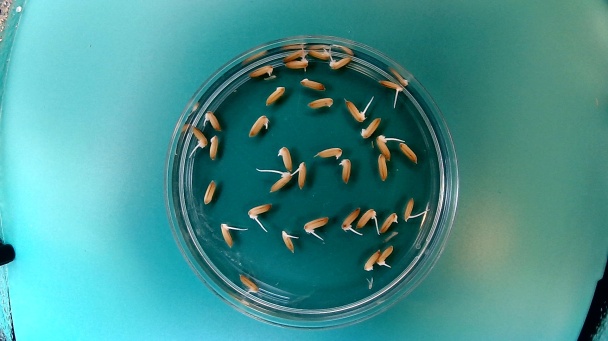 | 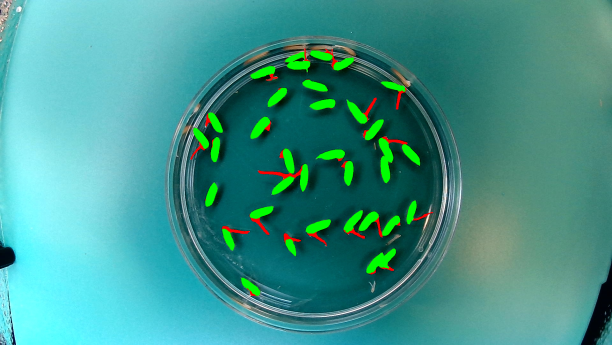 |
| 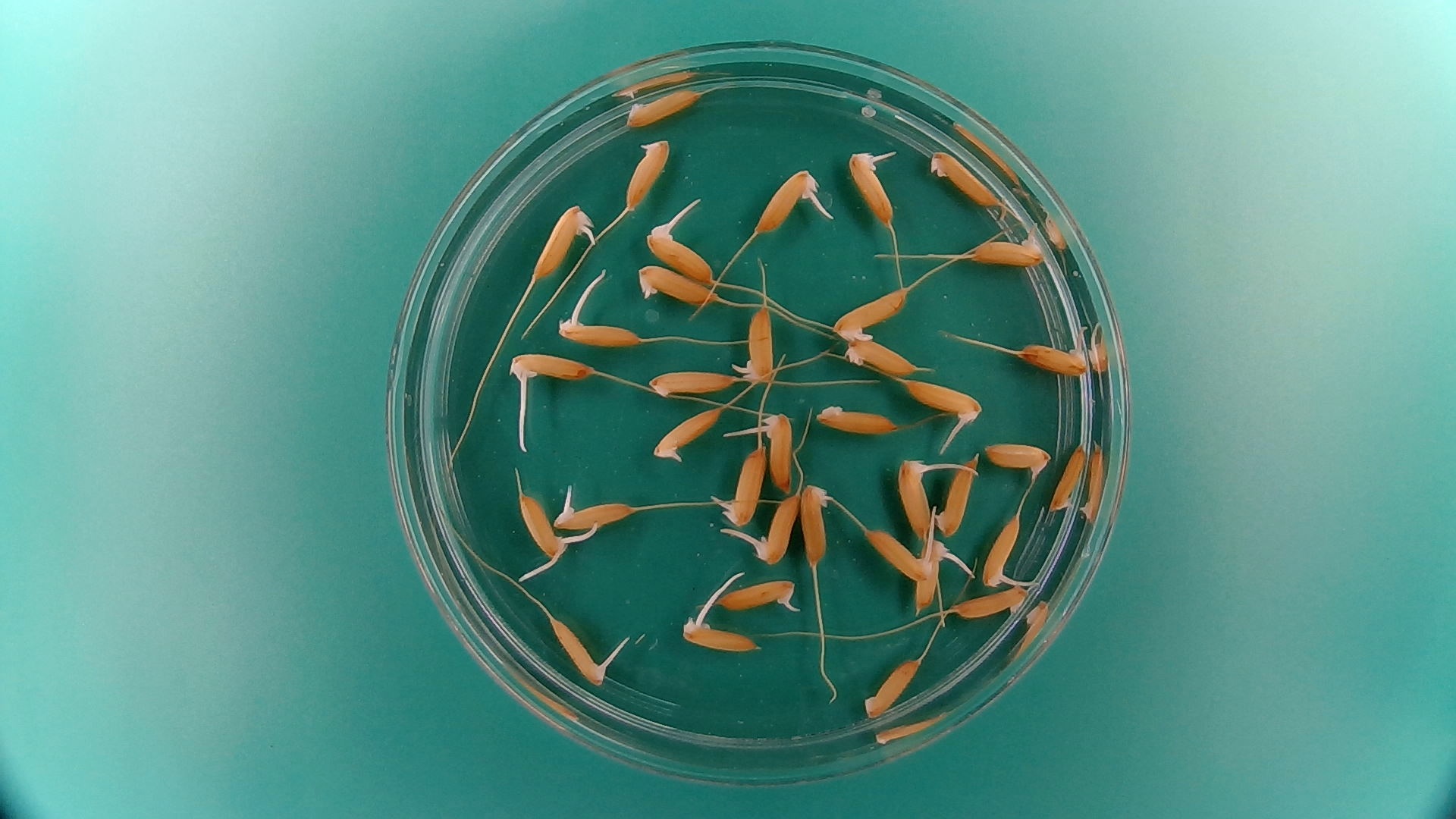 | 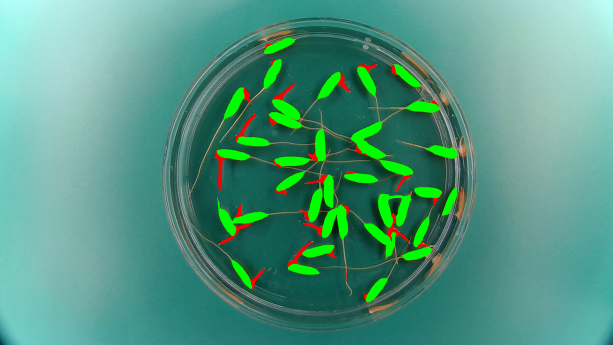 |
| 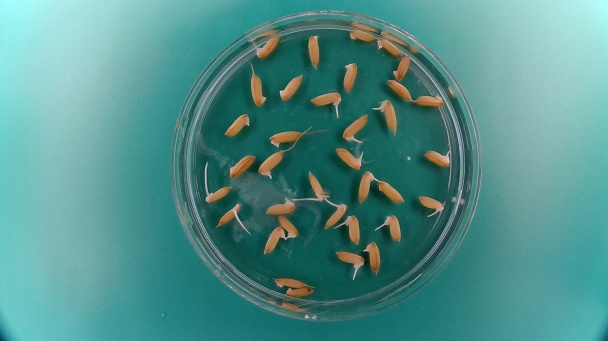 | 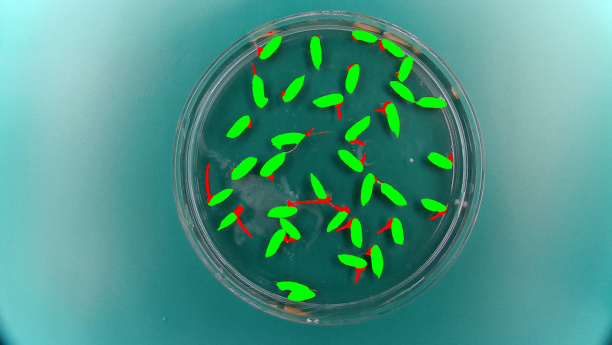 |
| 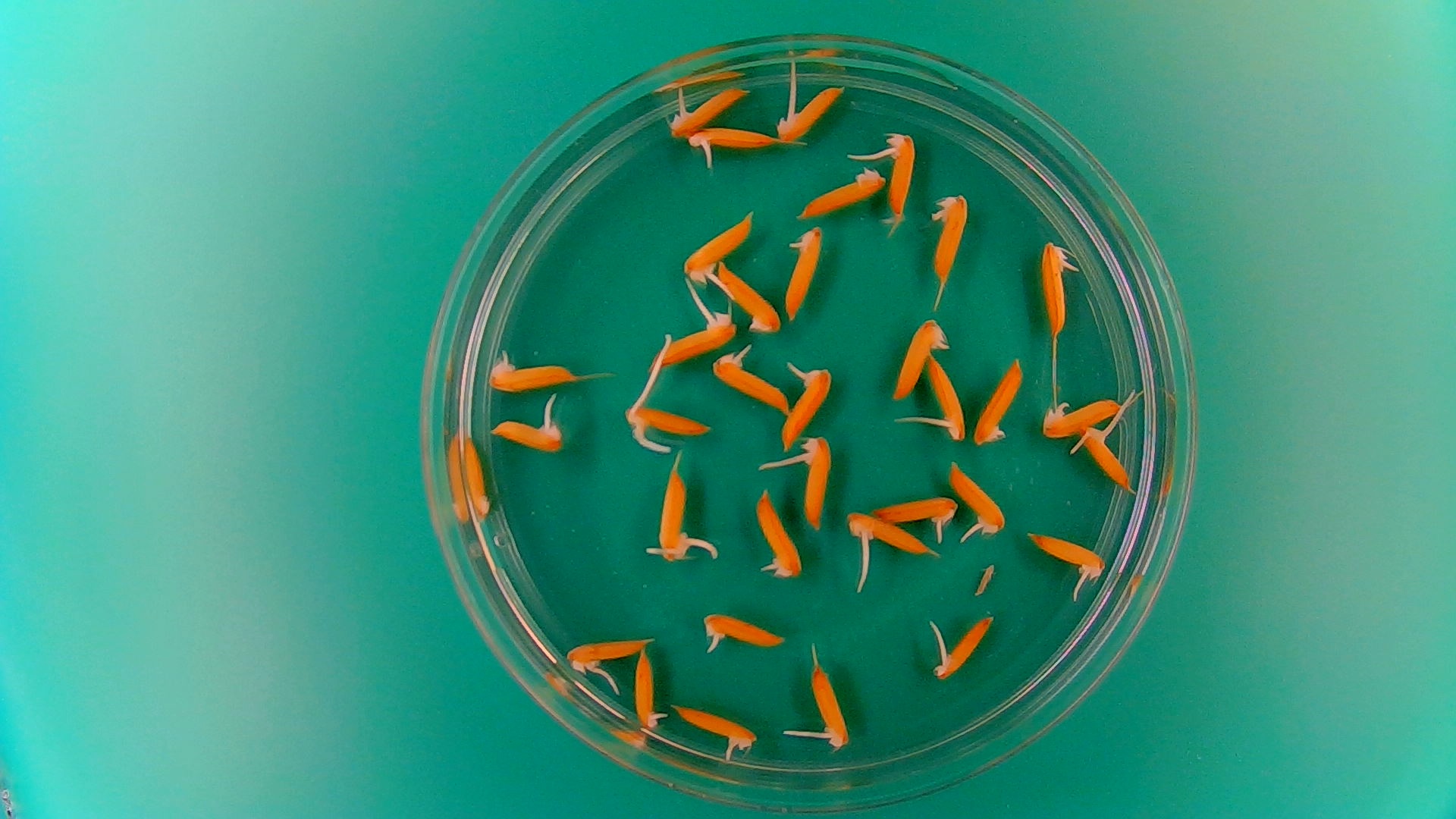 | 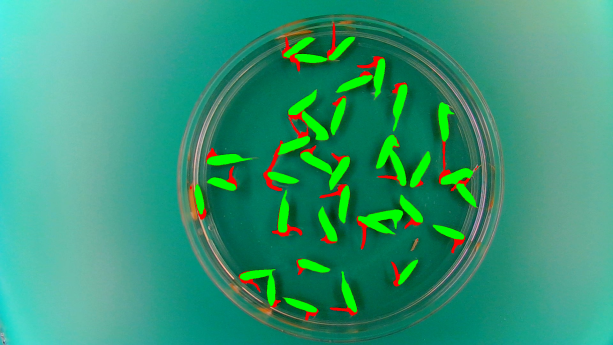 |
| 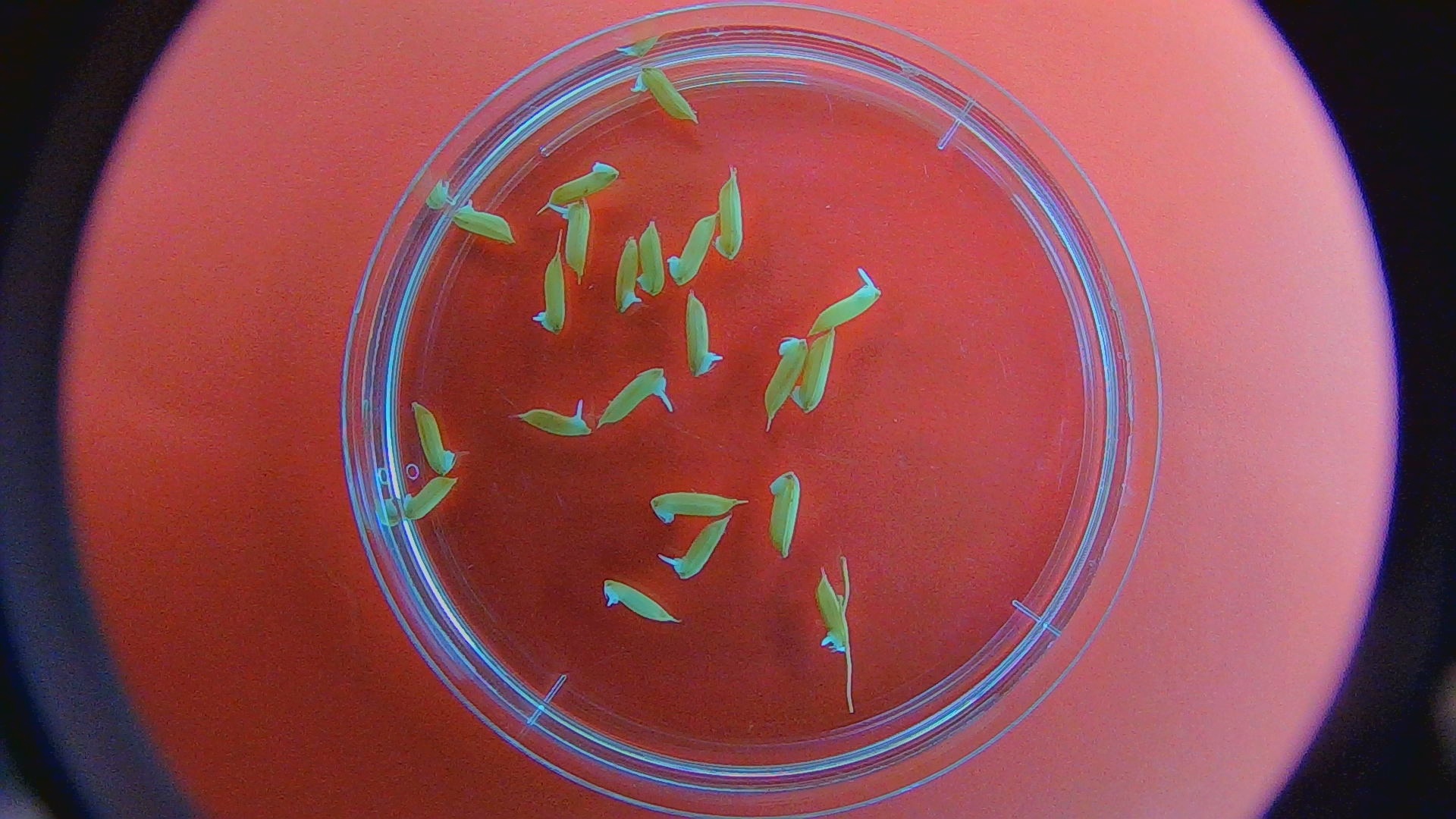 | 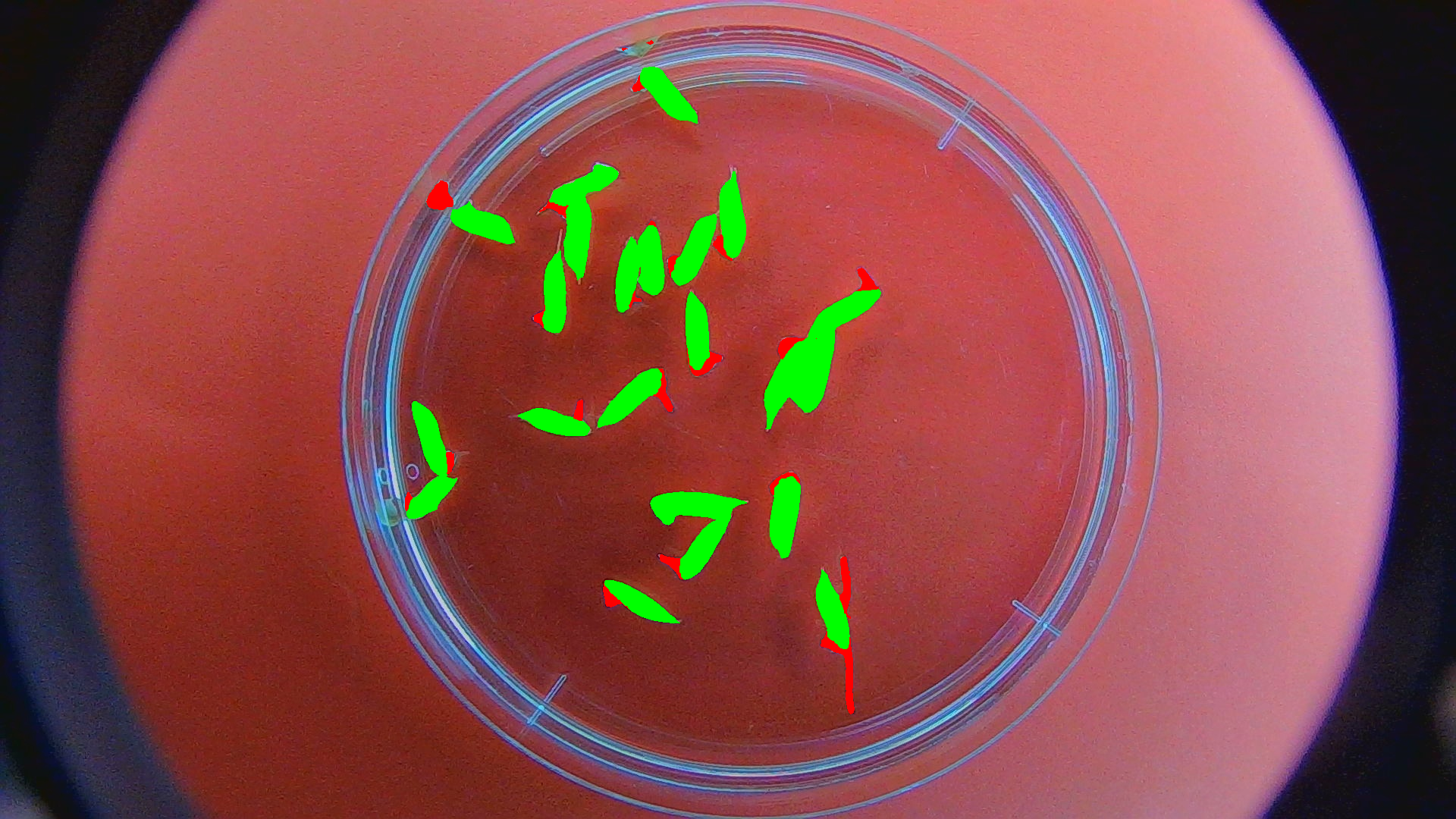 |

**Fig. S2.** Segmentation results under adhesion, shadow, and complex background conditions​​.


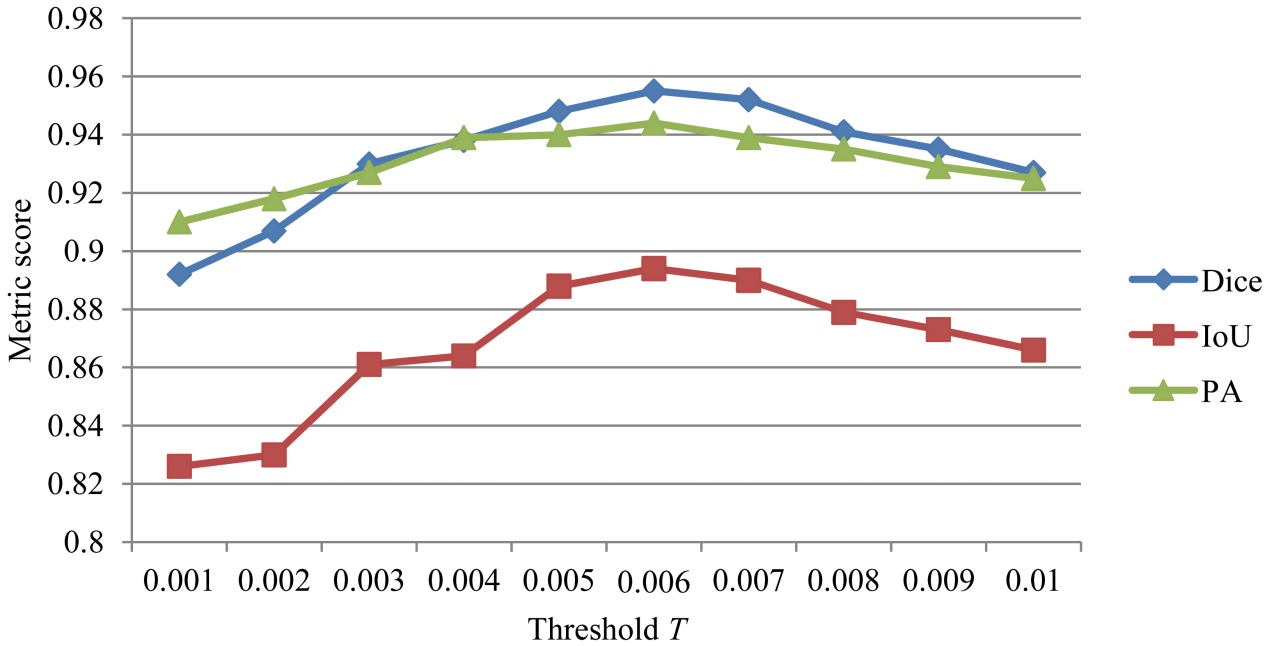


**Fig. S3.** Sensitivity of Segmentation Metrics to Threshold *T*.


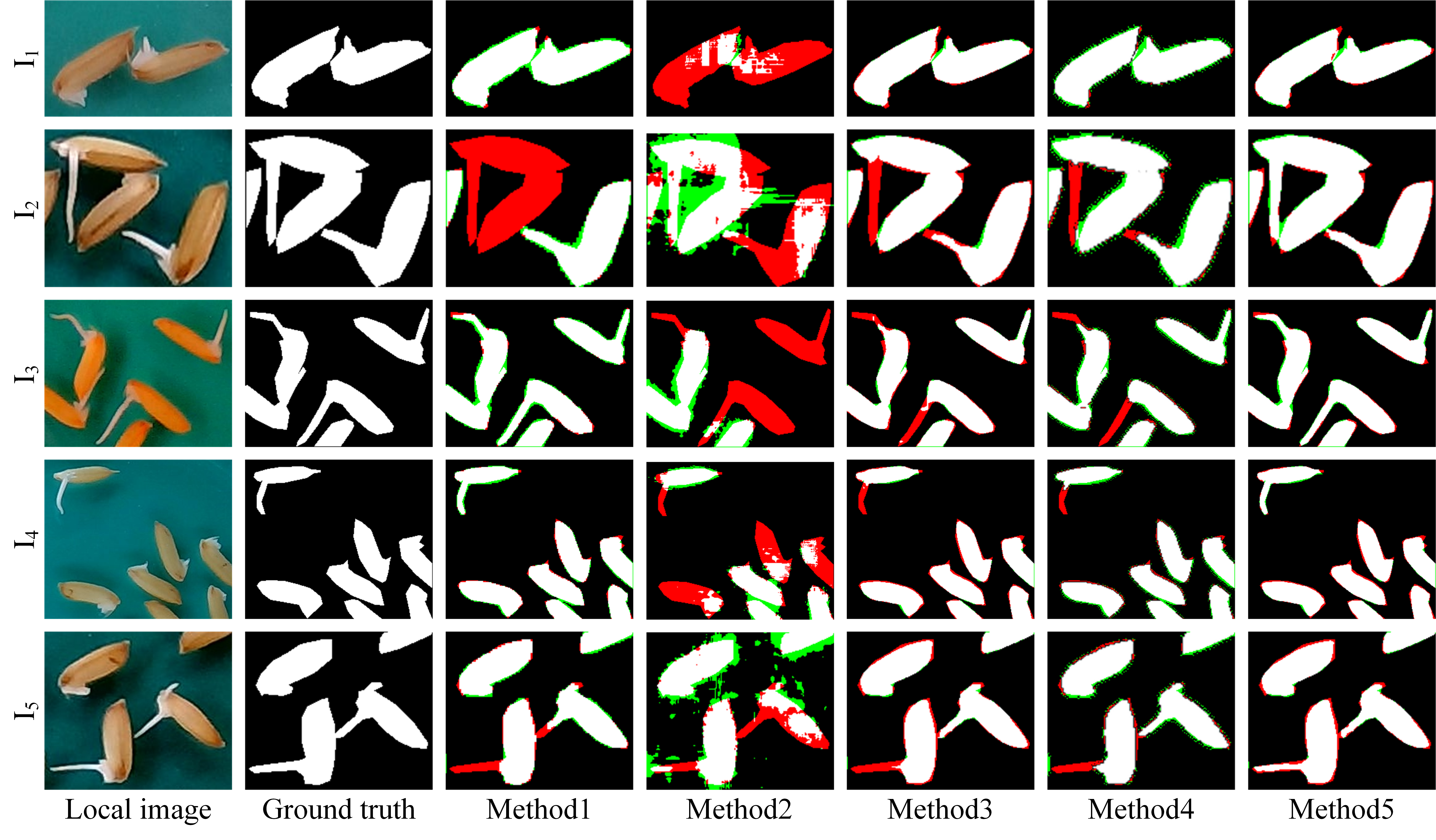


**Fig. S4.** The local segmentation results of the five algorithms on five images.


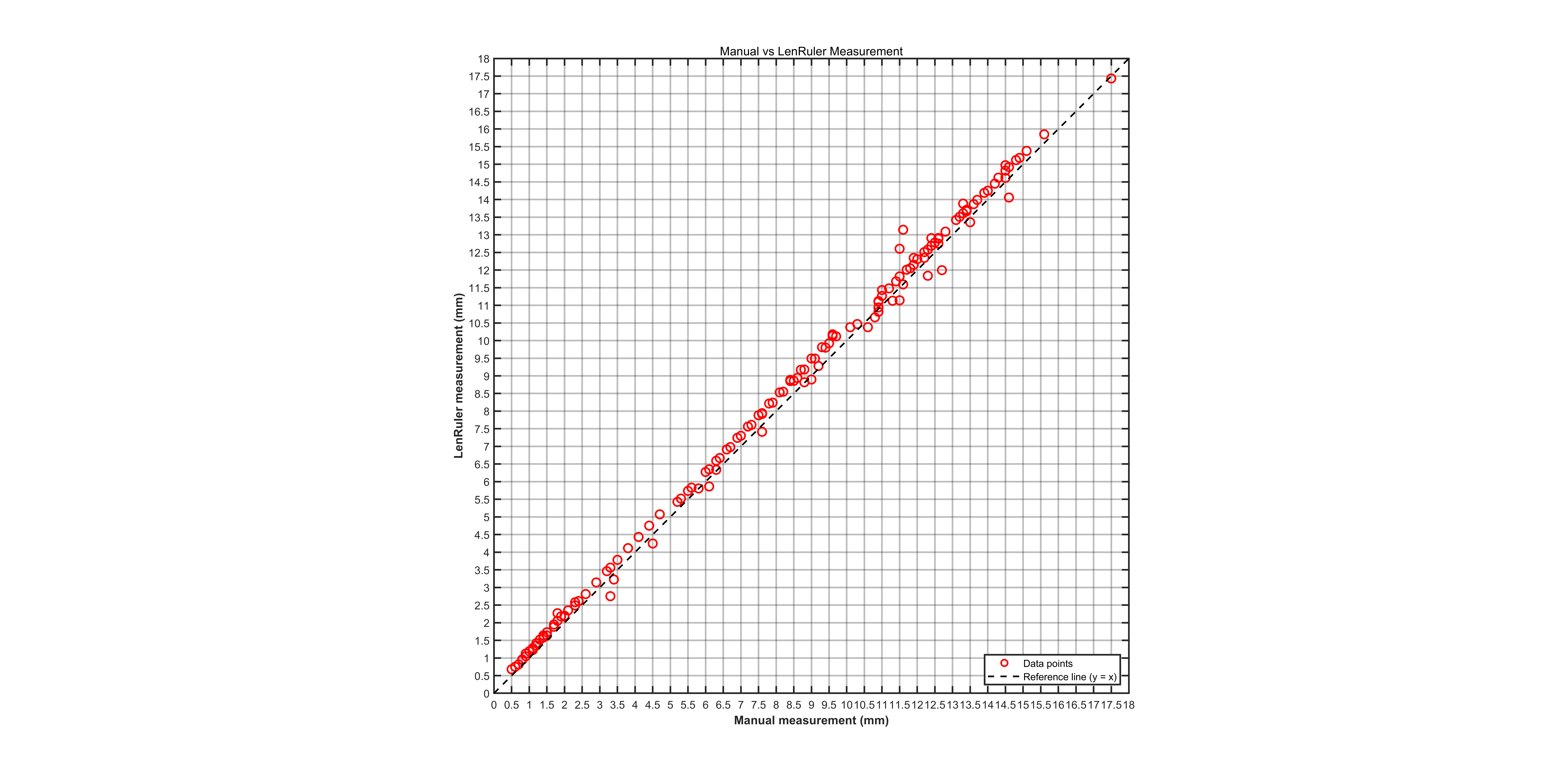


**Fig. S5.** Comparison of manual and LenRuler measurements for radicle length.
